# Anisotropic expansion of hepatocyte lumina enforced by apical bulkheads

**DOI:** 10.1083/jcb.202103003

**Published:** 2021-07-30

**Authors:** Lenka Belicova, Urska Repnik, Julien Delpierre, Elzbieta Gralinska, Sarah Seifert, José Ignacio Valenzuela, Hernán Andrés Morales-Navarrete, Christian Franke, Helin Räägel, Evgeniya Shcherbinina, Tatiana Prikazchikova, Victor Koteliansky, Martin Vingron, Yannis L. Kalaidzidis, Timofei Zatsepin, Marino Zerial

**Affiliations:** 1 Max Planck Institute of Molecular Cell Biology and Genetics, Dresden, Germany; 2 Department of Computational Molecular Biology, Max Planck Institute for Molecular Genetics, Berlin, Germany; 3 Skolkovo Institute of Science and Technology, Skolkovo, Russia; 4 Department of Chemistry, Lomonosov Moscow State University, Moscow, Russia; 5 Nelson Laboratories LLC, Salt Lake City, UT

## Abstract

Lumen morphogenesis results from the interplay between molecular pathways and mechanical forces. In several organs, epithelial cells share their apical surfaces to form a tubular lumen. In the liver, however, hepatocytes share the apical surface only between adjacent cells and form narrow lumina that grow anisotropically, generating a 3D network of bile canaliculi (BC). Here, by studying lumenogenesis in differentiating mouse hepatoblasts in vitro, we discovered that adjacent hepatocytes assemble a pattern of specific extensions of the apical membrane traversing the lumen and ensuring its anisotropic expansion. These previously unrecognized structures form a pattern, reminiscent of the bulkheads of boats, also present in the developing and adult liver. Silencing of Rab35 resulted in loss of apical bulkheads and lumen anisotropy, leading to cyst formation. Strikingly, we could reengineer hepatocyte polarity in embryonic liver tissue, converting BC into epithelial tubes. Our results suggest that apical bulkheads are cell-intrinsic anisotropic mechanical elements that determine the elongation of BC during liver tissue morphogenesis.

## Teaser

Belicova et al. report previously unrecognized apical membrane extensions, forming a pattern reminiscent of the bulkheads of boats, that determine the anisotropic expansion of hepatocyte lumina. Loss of the bulkheads caused by Rab35 silencing leads to re-engineering of epithelial polarity and liver tissue architecture.

## Graphical Abstract

**Figure:**
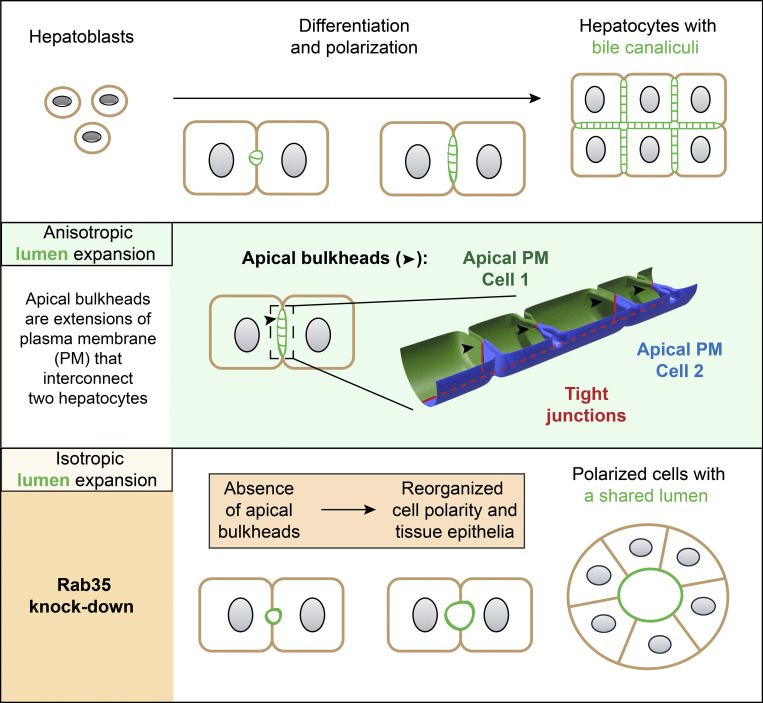

## Introduction

Lumen morphogenesis is essential for several organs. Lumina are generated by epithelial cells that exhibit apico-basal polarity, with the apical surface facing the internal lumen and the basal surface contacting the basement membrane (Bryant and Mostov, 2008; Andrew and Ewald, 2010; Sigurbjörnsdóttir et al., 2014). Lumina expand either isotropically, yielding spherical structures (acini and alveoli in vivo, cysts and organoids in vitro), or anisotropically, generating a variety of epithelial tube shapes across tissues (e.g., lungs, intestine, kidney, and liver). The anisotropic expansion of lumina is more difficult to explain than the isotropic one because it results from specific combinations of molecular pathways and physical forces (Datta et al., 2011; Navis and Nelson, 2016; Jewett and Prekeris, 2018; Dasgupta et al., 2018; Stopka et al., 2019; Duclut et al., 2019). Physical forces can act on the tissue or cellular level. The liver provides a good example for a variety of lumen morphogenesis that is essential for its function (Treyer and Müsch, 2013; Müsch, 2018; Ober and Lemaigre, 2018; Tanimizu and Mitaka, 2017; Gissen and Arias, 2015). It contains two types of epithelial cells, bile duct cells (cholangiocytes) and hepatocytes, both derived from embryonic progenitors called hepatoblasts (Müsch, 2018). Bile duct cells have the typical apico-basal polarity that can be described with one vector pointing to the apical surface (Morales-Navarrete et al., 2019; Scholich et al., 2020). The apical surface expands isotropically to form 3D cysts in vitro (Tanimizu et al., 2007; Prior et al., 2019), or generate tubes in vivo (Antoniou et al., 2009; Tanimizu et al., 2016), which elongate under tissue-level forces (Navis and Nelson, 2016). In contrast, hepatocytes have a complex polarity, where the apical surface elongates anisotropically as a tubular belt surrounding the cells (Morales-Navarrete et al., 2015). Such polarity can be described by biaxial nematic tensors, a mathematical description of the belt-like and multi-polar apical surface (Morales-Navarrete et al., 2019; Scholich et al., 2020). Hepatocytes can initiate apical lumina with multiple neighboring hepatocytes concurrently in all directions, allowing them to form a complex 3D luminal network of highly branched ∼1-µm-thin bile canaliculi (BC; Morales-Navarrete et al., 2015). The morphology of BC implies that the anisotropy of lumen elongation is not imposed by tissue-level forces but rather by local forces at the cellular level.

The mechanisms underlying the anisotropy of lumen formation at the cellular scale remain elusive. Models based mainly on in vitro studies propose cell division as a key determinant of lumen elongation (Wang et al., 2014; Tanimizu and Mitaka, 2017; Overeem et al., 2015). In the developing liver however, as hepatoblasts differentiate into hepatocytes, they gradually stop proliferating (Yang et al., 2017). Yet an almost fully connected BC network is generated (Tanimizu et al., 2016), arguing for additional mechanisms driving lumen elongation. From the physics of thin shells (Landau and Lifshitz, 1986; Berthoumieux et al., 2014), lumen elongation by fetal hepatocytes requires mechanisms based either on the anisotropic structure of the apical actomyosin cortex or some other mechanical elements to enforce a tubular lumen. Here, we set out to identify such mechanisms.

## Results

### Anisotropic lumen morphogenesis by hepatocytes

**Figure 1.**
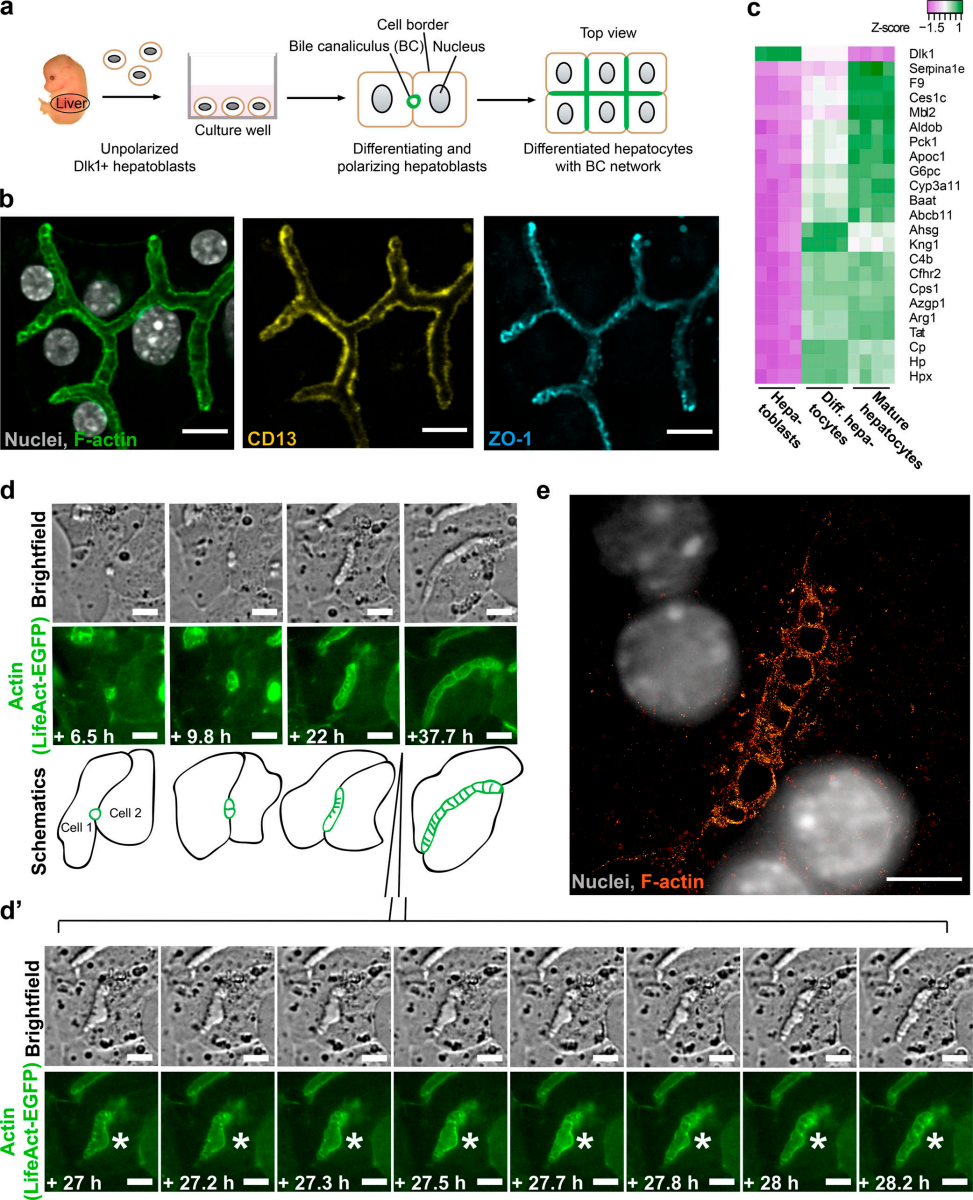
**Lumen morphogenesis in hepatocytes is accompanied by specific actin structures that interconnect the two lumen-forming cells. (a)** Schematic overview of primary Dlk1^+^ hepatoblasts in culture differentiating into hepatocytes and recapitulating BC formation. **(b)** Differentiated hepatocytes form branched interconnected BC lumina. Immunofluorescence microscopy images of differentiated hepatocytes (Protocol 2, Materials and methods) stained for F-actin with phalloidin–Alexa 488, and for apical markers CD13 and ZO-1. Scale bar: 10 µm. **(c)** In vitro differentiated (Diff.) hepatocytes express mature hepatocyte markers and down-regulate the hepatoblast marker Dlk1. Heatmap comparing the expression of selected hepatocyte marker genes in primary Dlk1^+^ hepatoblasts (Hepatoblasts), in vitro differentiated hepatocytes (Diff. hepatocytes, Protocol 1, Materials and methods), and control mature hepatocytes isolated from adult mouse livers (Mature hepatocytes). RNA-seq experiment in four biological replicates. **(d)** Images from live-cell time-lapse microscopy documenting the formation of BC between two differentiating hepatoblasts expressing LifeAct-EGFP. During imaging, the extending tubular lumen displayed a bulge at 27 h from the start of imaging, which was subsequently “reabsorbed.” The insert d’ documents the recovery of the tubule (white star) in temporal resolution 10 min/frame. Note the transverse striped pattern in brightfield and actin channels, which is apparent when the lumen is tubular but not observed within the bulge. Scale bar: 10 µm. See also Video 1. **(e)** SMLM image of a lumen between two differentiated hepatocytes, actin labeled with phalloidin–Alexa 647. Note the transverse striped actin pattern. Scale bar: 5 µm.

We established a culture of primary mouse hepatoblasts isolated from embryonic livers based on Dlk1 expression (Tanimizu et al., 2003) to differentiate them into hepatocytes. The differentiation was validated by the expression of mature hepatocyte markers and the acquisition of characteristic hepatocyte morphology (Fig. 1, a–c). The cells generated elongated and branched tubular lumina enriched in F-actin, and positive for the apical marker CD13 and the tight junction (TJ) protein ZO-1 (Fig. 1 b). This system therefore recapitulates de novo formation of branched BC lumina in vitro similar to the developing liver in vivo.

**Figure 2.**
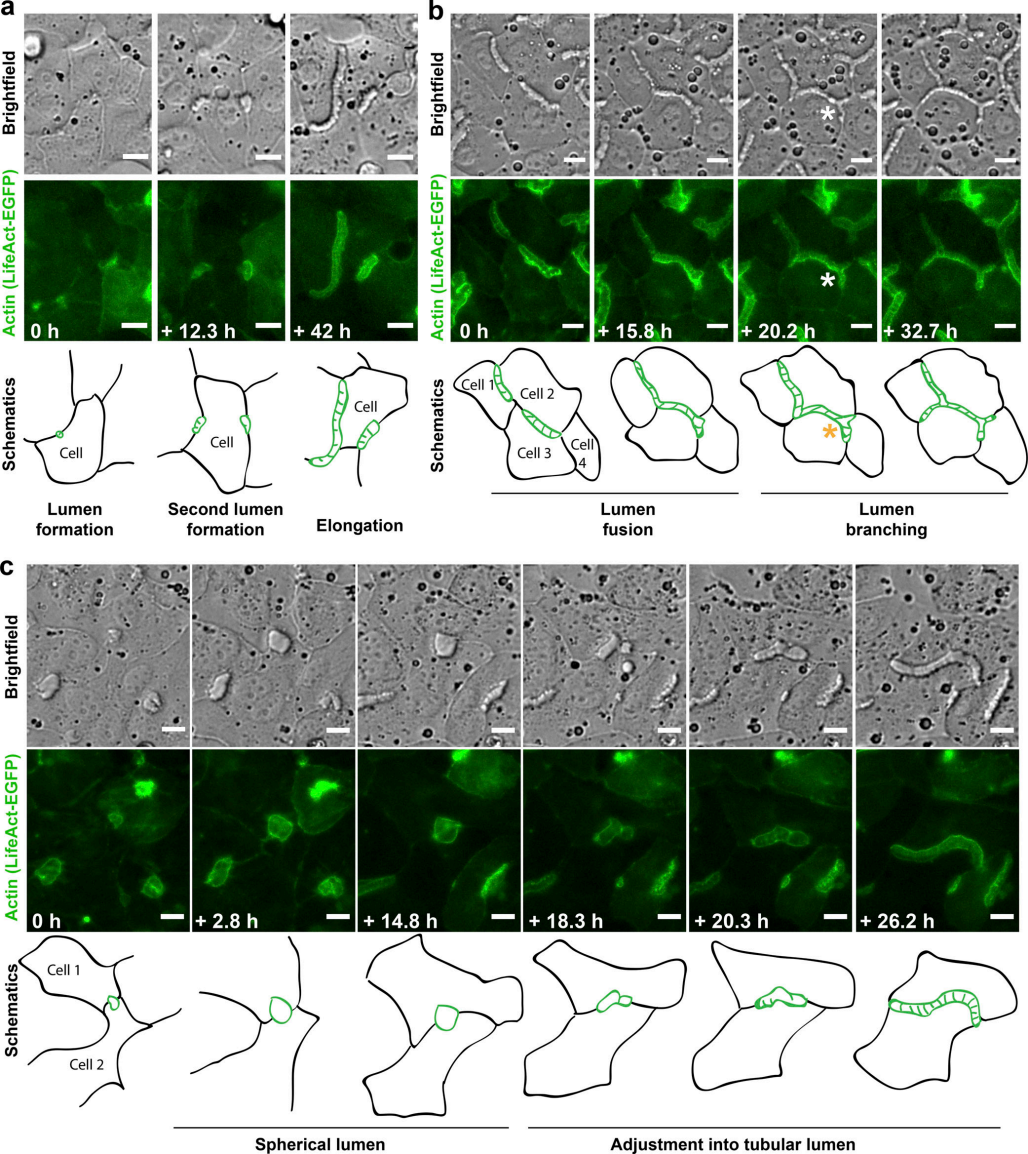
**Live-cell video microscopy of BC morphogenesis in LifeAct-EGFP–expressing cells. (a)** A single polarizing differentiating hepatoblast forming multiple tubular lumina. Scale bar: 10 µm. See also Video 2. **(b)** BC network forms between neighboring cells by fusion and branching (star) of elongated tubular lumina. Scale bar: 10 µm. See also Video 3. **(c)** A lumen formed between two cells starts growing spherically, but is later adjusted and continues to elongate as a typical BC lumen. Scale bar: 10 µm. See also Video 4.

To study lumen morphogenesis, we performed live-cell time-lapse microscopy on differentiating hepatoblasts stably expressing LifeAct-EGFP as actin label (Fig. 1 d and Video 1). We followed lumenogenesis for up to 52 h and categorized four sequential steps: (1) lumen initiation, (2) elongation, (3) branching, and (4) fusion. We frequently observed single cells initiating multiple individual lumina with their neighbors (Fig. 2 a and Video 2). After formation, lumina elongated into tubes until they spanned the entire cell–cell contact (Fig. 1 d; Fig. 2, a–c; Video 1; Video 2; Video 3; and Video 4). At this point, a lumen could fuse with another lumen (Fig. 2 b, left) or branch at a three-cell contact (Fig. 2 b, right). The elongation of lumina occurred in the absence of cell division.

**Video 1.** **Formation of BC in vitro*.*** Live-cell time-lapse microscopy documenting the formation of BC between two differentiating hepatoblasts expressing LifeAct-EGFP. Images acquired in 10-min intervals. The video displays a 23-h time window at 12,000× normal speed. Scale bar: 10 µm.

**Video 2.** **Formation of multiple BC lumina by a single differentiating hepatoblast.** Live-cell time-lapse microscopy documenting the formation of multiple BC lumina by a single differentiating hepatoblasts expressing LifeAct-EGFP. Images acquired in 10-min intervals. The video displays a 42-h time window at 12,000× normal speed. Scale bar: 10 µm.

**Video 3.** **Branching and fusion of BC lumen.** Live-cell time-lapse microscopy of branching and fusing BC lumina formed by differentiating hepatoblasts expressing LifeAct-EGFP. Images acquired in 10-min intervals. The video displays a 25-h time window at 12,000× normal speed. Scale bar: 10 µm.

**Video 4.** **Recovery of tubular BC lumen.** Live-cell time-lapse microscopy documenting the adjustment of a spherical lumen into a tubular lumen in differentiating hepatoblasts expressing LifeAct-EGFP. Images acquired in 10-min intervals. The video displays a 35-h time window at 12,000× normal speed. Scale bar: 10 µm.

We were intrigued by the presence of dark stripes in the bright-field, transverse to the direction of lumen elongation (e.g., Fig. 1 d and Fig. 2), which may correspond to high curvature of the apical membranes. The stripes also coincided with areas of high density of actin (LifeAct-EGFP). The pattern was evident early in lumen formation and continued as the lumina elongated, keeping a characteristic spacing between stripes (Fig. 1 d and Fig. 2). Interestingly, we also observed instances when the lumen transiently bulged outward, tending to a spherical lumen (Fig. 1 d’ [marked with a star], Video 1, Fig. 2 c, and Video 4). This coincided with the loss of the stripes. Subsequently, the tubular shape of the lumen recovered as new stripes formed, suggesting an active link between the striped pattern and lumen elongation.

To determine the micro-structure of the actin-rich stripes, we analyzed the cortical F-actin labeled with phalloidin–Alexa 647 using single-molecule localization microscopy (SMLM) on fixed, in vitro differentiated hepatocytes. Strikingly, we observed a quasi-periodic pattern of F-actin structures apparently crossing the lumen between two cells (Fig. 1 e), similar to the pattern of stripes in the bright-field (Fig. 1 d). Because the SMLM has a z-resolution of ∼500 nm and these structures are >1 µm in height, we can conclude that the F-actin projects into the BC lumen and does not correspond to rings around it, e.g., as in the *Drosophila *tracheal tube (Hannezo et al., 2015; Hayashi and Dong, 2017).

### Ultra-structural analysis reveals bulkhead-like apical transversal structures in the BC lumen

**Figure 3.**
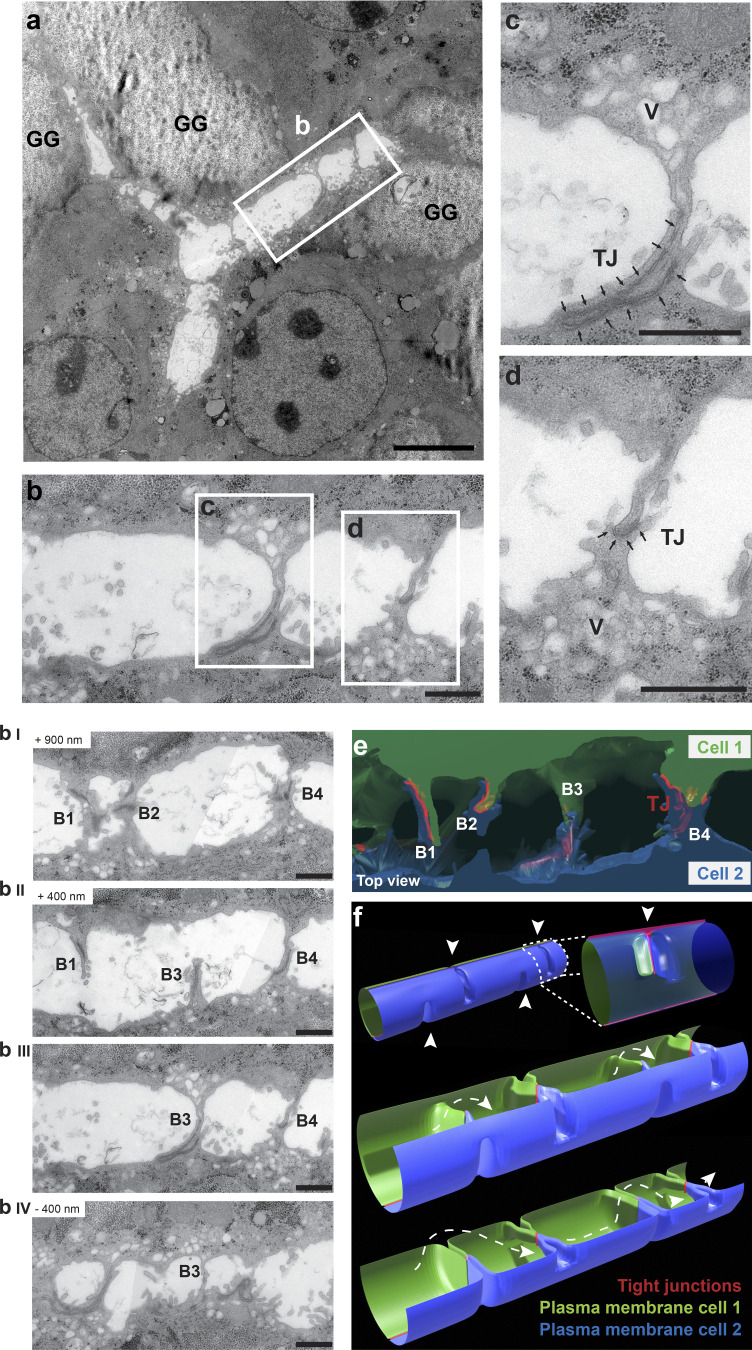
**Ultra-structural analysis reveals a bulkhead-like pattern of transversal structures sealed by T-shaped TJs in the BC lumen. (a)** EM image of a BC branched between three cells. Longitudinal section of in vitro differentiated hepatocytes. GG, glycogen granules. The outlined region is shown in b. Scale bar: 5 µm. **(b)** Section of BC formed by two cells with membrane connections highlighted in rectangles c and d and shown in detail in corresponding panels c and d. The sections were acquired in a series of longitudinal 90-nm sections shown in b, I–IV. Note that the membrane connections are not visible in every section. Scale bar: 1 µm. **(c and d)** Detailed view on the membrane connections in rectangles c and d from panel b. Membrane connections are formed by apical surfaces of both cells lining the BC and include the TJs (arrows). Vesicles (V) are often observed to accumulate in the vicinity of the connections. Scale bar: 1 µm. **(e)** 3D reconstruction of serial sections in b (I–IV) based on apical plasma membranes and TJs rendering. The cytoplasm of the lumen-forming cells is in green and blue, and the TJs are highlighted in red. See also Video 5. **(f)** Simplified model of BC based on the 3D reconstruction in e with periodic bulkhead-like membrane connections formed from the top or the bottom of the lumen (arrowheads). The TJs (red) have a T-shape, with the junctions longitudinal along the tube connected with the junctions extending along the ridgeline within each bulkhead. Uninterrupted flow within the lumen between bulkheads is shown with a dotted line. See also Video 6.

Given the presence of both actin filaments and TJs (ZO-1) traversing the lumen, we investigated these structures in greater detail by EM on serial sections and 3D reconstructions of the entire lumen volume. Remarkably, the EM section of Fig. 3 a shows a branched lumen between three hepatocytes, whose surfaces are connected by finger-like membrane processes. The fingers of one cell touch, or invaginate into, the opposing cell (Fig. 3 b), and the contact surfaces are sealed by TJs (Fig. 3, c and d). Interestingly, we often observed vesicles accumulated at the base of these processes.

From a single section it is impossible to establish whether the lumen is continuous or divided into separate chambers. The 3D reconstruction (Fig. 3 e and Video 5) revealed that the transversal finger-like processes (Fig. 3 a) were not microvilli but sections of structures resembling the bulkheads of a boat. The bulkheads consisted of two parts, each contributed by the apical surface of one of the two adjacent cells, which formed a ridge-shaped process (see 3D model, Fig. 3 f, Video 5, and Video 6). Importantly, the two ridges were sealed by TJs that followed an unusual T-shape, with the horizontal bar representing the junctions longitudinal along the tube and the vertical bar the junctions extending along the ridgeline (see scheme in Fig. 3 e, Video 5, and Video 6). The EM data are consistent with the presence of ZO-1 structures in the stripes crossing the lumen (Fig. 1 b). In some cases, the opposing processes are not precisely aligned along the ridgeline but shifted, forming a wide TJ contact belt (Fig. 3, b and e, bulkheads B1 and 4). The bulkheads can come either from the bottom (see Fig. 3 b, II–IV, and Fig. 3 e, bulkhead B3) or the top of the tube (Fig. 3 b, I–IV; and Fig. 3 e, bulkheads B1, 2, and 4), but never separate it completely, thus ensuring lumen continuity in the BC (Video 5 and Video 6). Consequently, from the 3D reconstruction of Fig. 3 b and Video 5, one can appreciate that the lumen has a tortuous shape. This accounts for the impression that the F-actin fluorescent and bright-field stripes only partially cross the lumen (Fig. 1 d). The bulkheads showed a quasi-periodicity similar to the pattern in the bright-field and of actin observed by live-cell imaging (Fig. 1 d) and SMLM (Fig. 1 e).

**Video 5.** **3D model of BC based on EM data.** 3D reconstruction of EM serial sections in Fig. 2 b based on apical plasma membranes and TJs rendering. The apical plasma membrane of the lumen-forming cells is in green and blue, the TJs are highlighted in red.

**Video 6.** **Animation of a simplified model of BC with bulkheads.** Simplified model of BC based on the 3D reconstruction in Fig. 2 e with periodic bulkhead-like membrane connections containing TJs (red) formed from the top or the bottom of the lumen. The apical plasma membrane of the lumen-forming cells is represented in green and blue.

In summary, the apical bulkheads are morphologically distinct from the finger-like shaped microvilli and are not simply folds of the BC. They (1) are membrane processes from two opposing cells sealed by TJs, (2) have a plate/ridge-like 3D shape, and (3) do not separate the lumen in distinct chambers.

### Apical bulkheads form during BC lumen morphogenesis in embryonic liver and persist in adulthood

**Figure 4.**
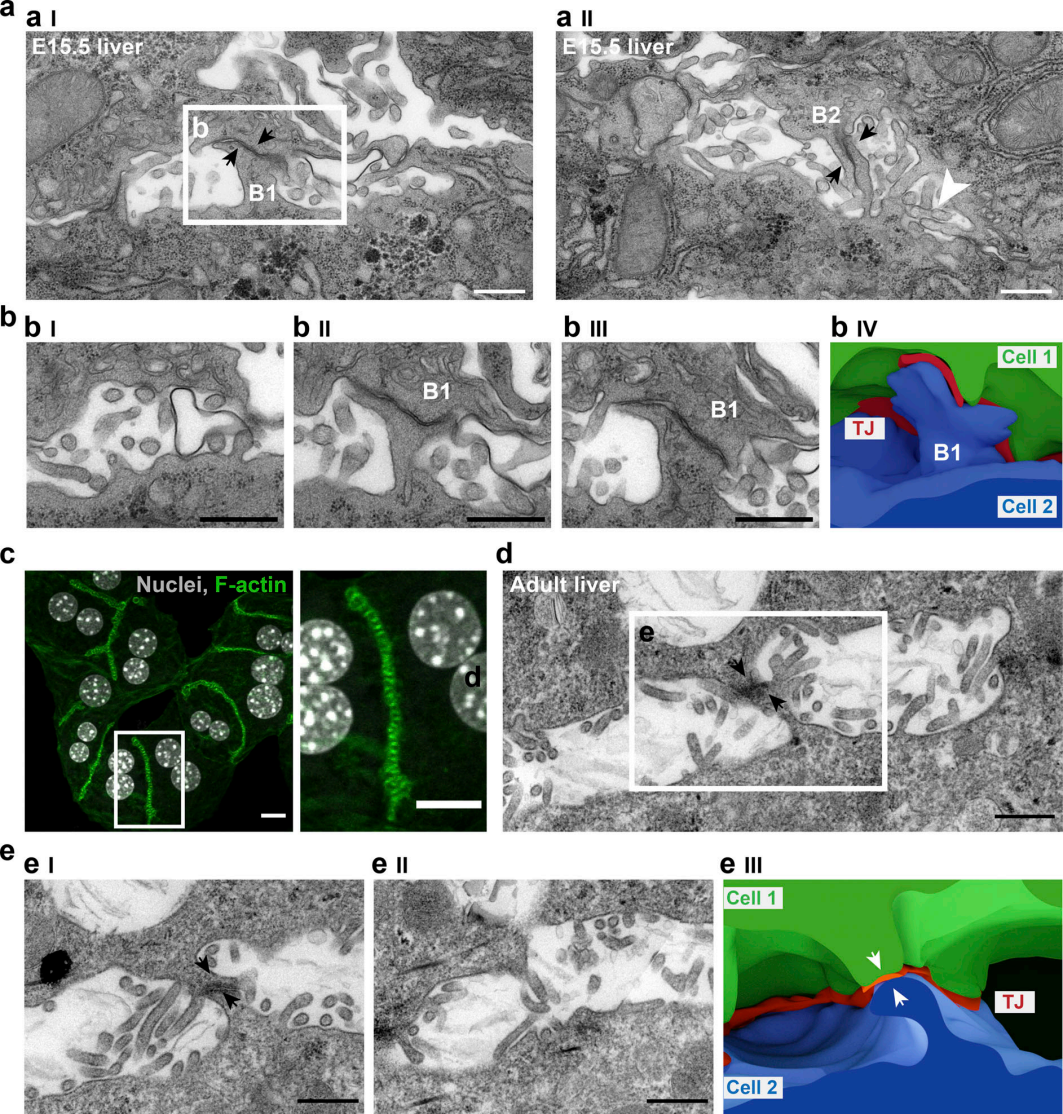
**Transversal apical membrane structures form during BC lumen morphogenesis in embryonic liver and persist in adulthood. (a)** EM images of two serial sections (I and II) of a forming BC in a E15.5 liver. The black arrows point to the TJs within the bulkhead-like membrane connections B1 and B2. Scale bar: 500 nm. **(b)** Serial sections (I–III) focusing on bulkhead B1 show that the bulkhead does not separate the lumen completely. The lumen is continuous in section I, and the bulkhead appears above in sections II and III. Scale bar: 500 nm. IV, 3D reconstruction of the bulkhead B1 from the serial sections. **(c)** Fluorescent microscopy images of cultured mature hepatocytes isolated from adult livers. F-actin staining by phalloidin shows a striped pattern similar to that in Fig. 1 b. Scale bar: 10 µm. **(d)** EM image of BC in an adult liver section. Black arrows point to the TJs in the bulkhead. Scale bar: 500 nm. **(e)** Serial sections of the bulkhead in d. The bulkhead protrudes into the lumen but does not separate it, as the lumen in II is continuous. Scale bar: 500 nm. IV, 3D reconstruction of the bulkhead in d. White arrows point to the parts of bulkhead contributed by opposing cells connected via TJs.

To rule out that these structures are an artifact of the in vitro system, we examined the embryonic day (E) 15.5 liver by EM. Also here we could confirm the presence of the repetitive pattern of bulkhead-like transversal connections in the nascent BC (Fig. 4 a, bulkheads B1 and B2). The lumen shape was even more complex than in vitro, due to the 3D organization of the tissue, with a higher degree of freedom for cell–cell contacts. The 3D reconstruction of one bulkhead (B1) from the serial sections shows again the sealing of the two cellular processes by the TJs, which are continuous with the TJ belt along the BC (Fig. 4 b, IV). Importantly, also in vivo, the bulkheads did not divide the BC lumen into isolated chambers (Fig. 4 b, I). Compared with in vitro, microvilli were better preserved in vivo, and one can appreciate the morphological difference between microvilli and bulkheads (Fig. 4 a, II, white arrowhead pointing to a cluster of microvilli).

Similar to the differentiating hepatoblasts (Fig. 1, b, d, and e), apical bulkheads were also formed by primary mouse hepatocytes from adult liver in vitro (Fig. 4 c). Importantly, they were observed in 3D reconstructions of adult liver sections (Fig. 4, d and e), underscoring their physiological relevance.

### Conversion of hepatocyte biaxial polarity into vectorial polarity

**Figure S1.**
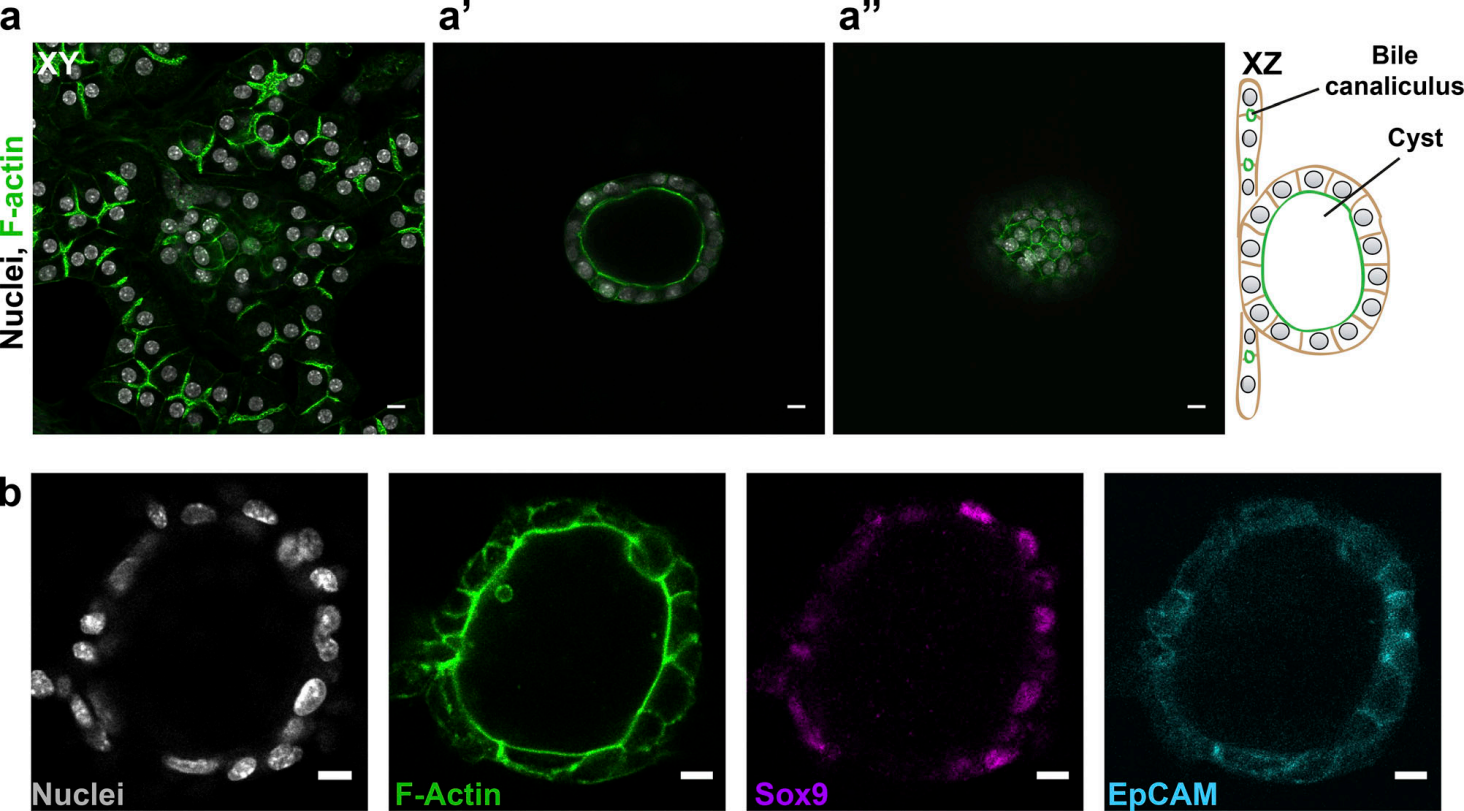
**Culture system supports the establishment of two types of epithelial polarity. (a)** A mixture of primary hepatoblasts and bile duct cells form BC and cysts under the same culture conditions. Images at different z-positions demonstrate that cysts grow in the z-direction (a’ and a’’), while hepatocytes with BC form a cell layer close to the well bottom. Cells stained for F-actin with phalloidin–Alexa 488. Scale bar: 10 µm. Schematics represent xz view. **(b)** Immunofluorescence staining for bile duct markers Sox9 and EpCAM in cyst-forming cells. Scale bar: 10 µm

The bulkhead-like apical processes could be a specific feature of hepatocyte polarity to enable the anisotropy of apical lumen growth. If so, their loss may convert hepatocyte biaxial polarity into vectorial epithelial polarity and induce the formation of cysts. Our in vitro system enables both types of polarity simultaneously, side by side in the same culture. The hepatoblasts that differentiate into hepatocytes form branched BC-like structures at the bottom of the well (Fig. S1 a), whereas the bile duct (Sox9^+^, EpCAM^+^) cells form 3D cysts rising into the medium (Fig. S1 a', a'', and b).

**Figure 5.**
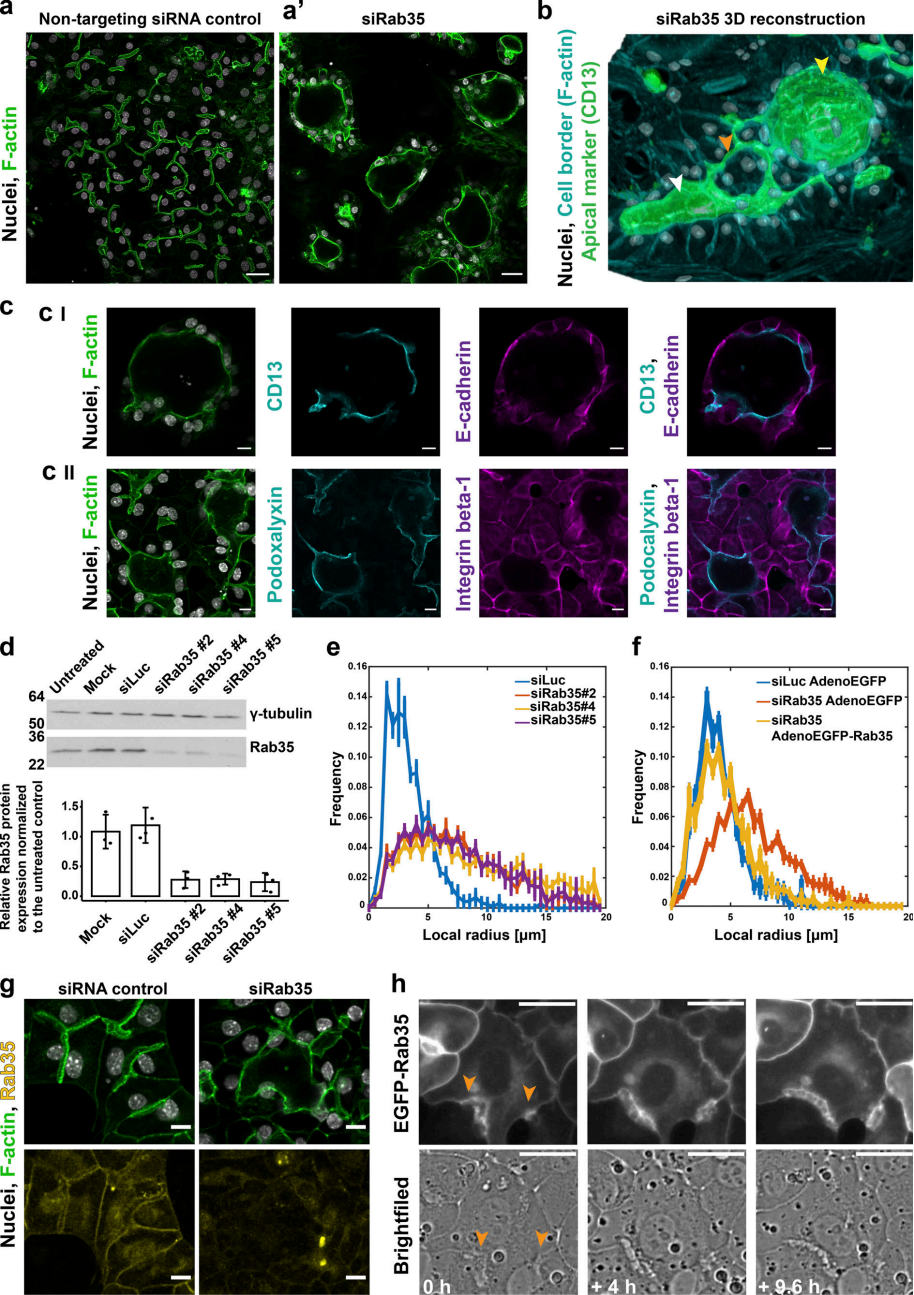
**Conversion of hepatocyte biaxial polarity into vectorial apico-basal polarity. (a)** Knock-down of Rab35 in differentiating hepatoblasts caused formation of cyst-like structures (a’), whereas cells treated with the control siRNA (siLuc) were unaffected and formed BC. Microscopy images of cells stained for F-actin with phalloidin–Alexa 488. Scale bar: 30 µm. See also Video 7. **(b)** 3D reconstruction of the cells treated with Rab35 siRNA show the variability of the phenotype from enlarged swollen lumina of epithelial tubes (white arrowhead) to spherical cyst-like structures (yellow arrowhead) growing in the z-direction (lumina stained with the apical marker CD13). Some remaining BC (orange arrowhead) connected to the cyst. **(c)** Localization of polarity markers in cyst-like structures upon Rab35 siRNA treatment. Microscopy images of cells immunostained for apically localized proteins CD13 (I) and podocalyxin (II) and basolaterally localized E-cadherin (I) and integrin β-1 (II). Scale bar: 10 µm. **(d)** Three independent Rab35 siRNA duplexes down-regulate Rab35 protein levels by 73% ± 3% (*n* = 3, error bars: SD). Representative Western blot and quantification of protein knock-down. **(e)** Histogram of the local lumen radius in control cells and cells treated with Rab35 siRNA estimated based on microscopy image analysis. Rab35 knock-down by three independent siRNAs results in the shift toward the lumina with larger radius. Percentage of lumina >6 µm: siLuc: 1.00% ± 0.46%, siRab35#2: 20.55% ± 1.66%, siRab35#4: 24.86% ± 1.46%, siRab35#5: 19.95% ± 2.81% (*n* = 3 [with four images per condition], error bars: SEM). **(f)** The enlarged lumina phenotype in the cells treated with Rab35 siRNA is rescued by expression of human Rab35-EGFP from recombinant adenovirus. The frequency curve of the lumen radius (yellow) overlaps with the one of the control cells expressing EGFP only (blue). EGFP alone does not affect the lumen enlargement caused by Rab35 knock-down (red). Percentage of lumina >6 µm: siLuc AdenoEGFP: 2.39% ± 0.44%, siRab35 AdenoEGFP: 22.35% ± 1.08%, siRab35AdenoEGFP-Rab35: 3.33% ± 1.05% (*n* = 3 [with four images per condition], error bars: SEM). **(g)** Microscopy images of differentiated hepatocytes treated with Luciferase or Rab35 siRNA stained with Rab35 antibodies (yellow). Rab35 localizes to the apical and lateral plasma membrane and cytoplasmic puncta. The levels of Rab35 are markedly reduced in the cyst-like structures formed upon Rab35 siRNA transfection. Scale bar: 10 µm. **(h)** Localization of exogenous EGFP-Rab35 in polarizing hepatoblasts. Selected images from live-cell time lapse microscopy. Two forming lumina are marked with orange arrowheads. Scale bar: 10 µm.

**Figure S2.**
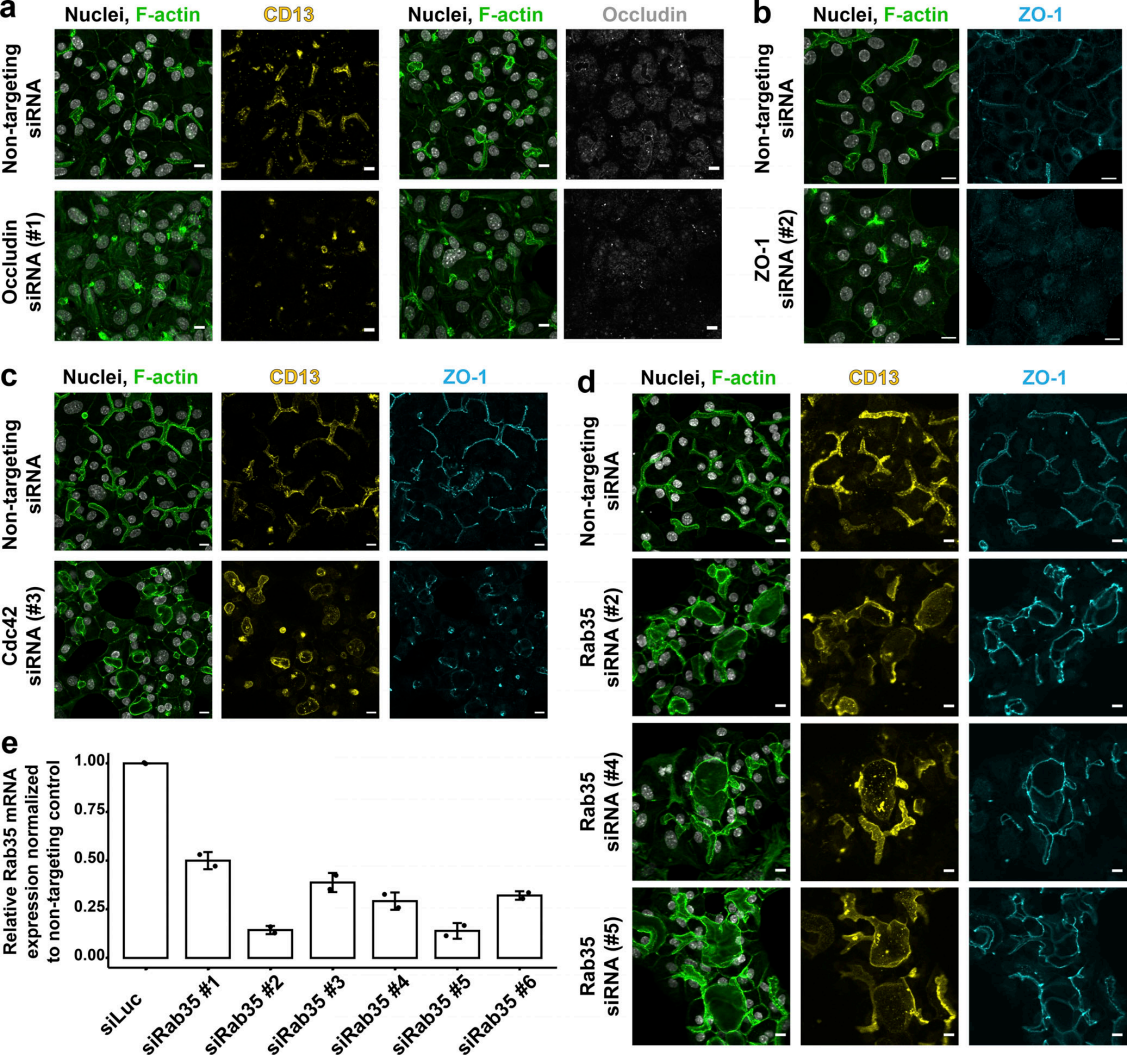
**Conversion of hepatocyte biaxial polarity into vectorial apico-basal polarity. (a)** Down-regulation of occludin impairs the lumen formation in differentiating and polarizing hepatoblasts. Immunofluorescence images of cells stained for the apical marker CD13 and occludin. Scale bar: 10 µm. **(b)** Down-regulation of the TJ protein ZO-1 impairs the lumen formation in differentiating and polarizing hepatoblasts. Immunofluorescence images of cells stained for ZO-1. Scale bar: 10 µm. **(c)** Down-regulation of Cdc42 leads to spherical lumina instead of BC in polarizing hepatoblasts. The polarity is not perturbed, as apical markers CD13 and ZO-1 still localized to the formed lumina. Scale bar: 10 µm. **(d)** Down-regulation of Rab35 leads to profound changes in lumen morphology of polarizing and differentiating hepatoblasts. The cells form multicellular structures with a shared lumen positive for apical markers CD13 and ZO-1. Immunofluorescence images of cells treated with three different siRNAs. Scale bar: 10 µm. **(e)** Estimation of knock-down efficiency of six siRNAs designed to target Rab35 mRNA 96 h post-transfection in differentiating and polarizing hepatoblasts in vitro (*n* = 2, SD).

Therefore, to identify genes required for hepatocyte polarity, we performed a focused siRNA screen on 25 candidate genes, encoding key regulatory components of cell polarity (Table S1): apical junction formation (e.g., Pard3, Tjp1, and Cldn2), cytoskeleton regulation (e.g., Mark2/Par1b, Stk11/Lkb1, and Cdc42), and polarized trafficking (e.g., Rab11a, Rab35, and Cdc42), including genes previously associated with the regulation of hepatocyte polarity (Wang et al., 2014; Fu et al., 2010; Cohen et al., 2004; Yuan et al., 2009). Hepatoblasts were transfected with the siRNAs and after 5 d in culture stained for F-actin, which is enriched at the apical domain (Fig. 1, b and d). Hit candidates were those yielding a penetrant lumen phenotype with a minimum of two siRNAs. Silencing of *Ocln* (Fig. S2 a) and *Tjp1* (Fig. S2 b) yielded a loss of cell polarity, with de-localized F-actin and the apical marker CD13 due to the absence of lumen. Remarkably, out of the 25 genes screened, down-regulation of Cdc42 and Rab35 did not disrupt cell polarity, as judged by the apical localization of CD13 and ZO-1, but altered lumen morphology (Fig. 5, a–c; and Fig. S2, c and d). *Cdc42* silencing caused dilated spherical lumina (Fig. S2 c); however, Rab35 knock-down yielded the most striking phenotype, causing the appearance of epithelial tubes (white arrowhead, Fig. 5 b) and large cyst-like structures (yellow arrowhead, Fig. 5 b) compared with control (Luciferase, siLuc; Fig. 5, a and b; and Fig. S2 d). In the cells forming the cysts, the apical markers CD13 and podocalyxin and basolateral markers E-cadherin and integrin β-1 also maintained their respective localization (Fig. 5 c), suggesting that knock-down of Rab35 also did not lead to inversion of polarity as reported for MDCK cells (Klinkert et al., 2016). Therefore, the depletion of Rab35 caused a change from biaxial to vectorial polarity (Morales-Navarrete et al., 2019; Scholich et al., 2020). Interestingly, the lumina of the tubes and cysts were connected with the residual BC (orange arrowhead, Fig. 5 b). Such a connection resembles morphologically the connection of BC to bile ducts, although the cells of the cysts were not bile duct cells as they were negative for the cholangiocyte marker Sox9.

### Rab35 is rate-limiting for the generation of hepatocyte lumina

Given the strength of the phenotype and because Rab35 had no previous connection to hepatocyte lumen morphology, we explored its function in more detail. To begin with, we validated the specificity of the Rab35 RNAi phenotype. First, out of six designed siRNAs, five yielded Rab35 mRNA down-regulation >50% after 96 h and showed various degrees of lumen alteration (Fig. S2, d and e). The three siRNAs (siRab35 #2, #4, and #5) that consistently yielded the strongest phenotype reduced Rab35 mRNA (Fig. S2 e) and protein level >70% (Fig. 5 d). Second, we rescued the Rab35 RNAi phenotype by expressing human Rab35, which is resistant to siRab35 #4. We quantified the effect of Rab35 knock-down on lumen morphology by measuring the radius of individual lumina in the control and knock-down conditions, and plotting the frequency distribution of values. There was a consistent shift toward larger lumina in the knock-down conditions by the three siRNAs targeting Rab35 mRNA (Fig. 5 e). Importantly, whereas in control conditions lumina barely had a radius >6 µm, upon Rab35 silencing, ∼20–25% of lumina had a radius >6 µm. Re-expression of human EGFP-Rab35 rescued the phenotype, shifting the distribution of lumen radius toward the control, whereas expression of EGFP had no affect (Fig. 5 f).

Rab35 was enriched in the apical surface as well as lateral plasma membrane and cytoplasmic vesicles (Fig. 5 g), in line with its endosomal localization (Kouranti et al., 2006; Klinkert et al., 2016). Upon silencing, this staining was markedly reduced (Fig. 5 g). Expression of exogenous EGFP-tagged Rab35 yielded a similar pattern of localization (Fig. 5 h). During lumen formation, EGFP-Rab35 was not only enriched apically but also present on the transversal connections, which were dynamically remodeled as the apical lumen expanded anisotropically.

### Loss of apical bulkheads and cyst formation upon Rab35 knock-down via cell self-organization

**Figure 6.**
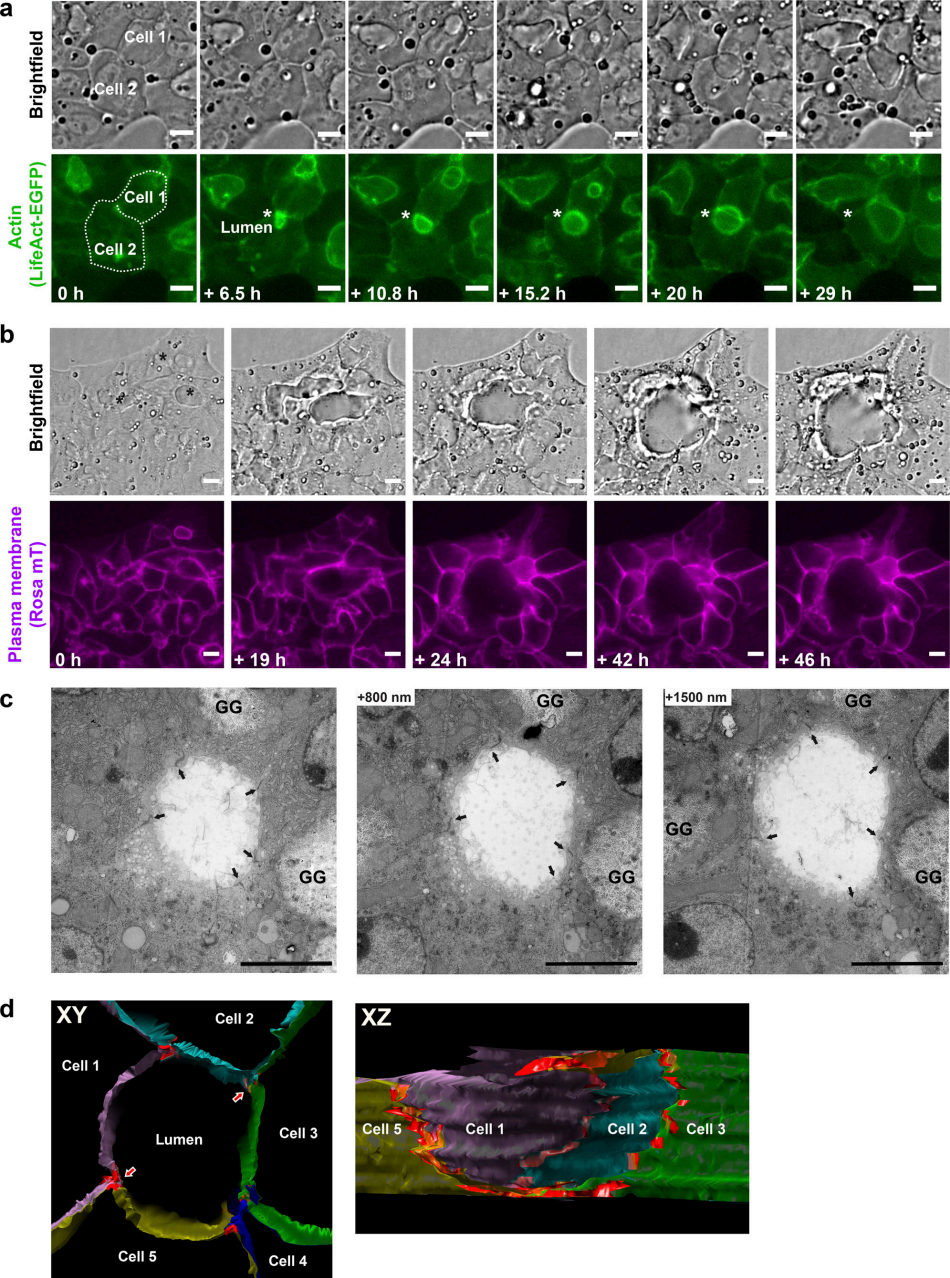
**Silencing of Rab35 causes the loss of the transversal apical membrane bulkheads and formation of spherical cysts via a cell self-organization process. (a)** In the cells treated with Rab35 siRNA, the lumina tend to grow as spheres instead of elongating as tubes. Images from the live-cell time-lapse microscopy experiment showing two neighboring differentiating hepatoblasts expressing LifeAct-EGFP under Rab35 siRNA conditions. The white star indicates the forming lumen between the two cells. Note that the typical transverse striped actin pattern observed in the tubular BC is absent. Scale bar: 10 µm. See also Video 8. **(b)** Multicellular cyst-like structures form by cell rearrangements. Images from the live-cell time-lapse microscopy experiment. The cells self-organize in such a way that the three separate lumina (black star) eventually fuse into one large spherical lumen, in the absence of cell division. Scale bar: 10 µm. See also Video 9. **(c)** EM analysis of a cyst-like lumen resulting from the Rab35 knock-down. A series of longitudinal 90-nm sections of the lumen formed between five cells. The bulkheads typical for hepatocyte BC are absent in the lumen. Arrows indicate TJs. GG, glycogen granules. Scale bar: 5 µm. **(d)** Longitudinal view (left) through the middle of a 3D model of the lumen based on rendering plasma membranes and TJs (red) on serial sections. The five cells forming the lumen are represented in different colors. Red arrows point to the TJs at which the cyst is cut open to reveal the sagittal view (right). The lumen has a circular profile, and TJs do not protrude into the lumen.

To gain insights into the change in polarity and lumen morphogenesis, we imaged LifeAct-EGFP expressing cells transfected with Rab35, or Luciferase siRNA as control, by live-cell time-lapse microscopy. Whereas normal and control differentiating hepatoblasts formed elongated lumina (e.g., Fig. 1 d), upon Rab35 depletion, they generated spherical lumina, initially between two cells (Fig. 6 a and Video 7). With time, we observed major cell rearrangements, whereby cells moved and reshaped their apical surface, leading to the fusion of lumina and the formation of 3D multicellular cysts (Fig. 6 b and Video 8), similar to Fig. 5 b. Again, such a reorganization was not a result of cell division, as for other cysts formed in vitro (Jewett and Prekeris, 2018), but rather by a self-organization process. A spherical expansion of the lumen occurred only in the cases where the cells failed to form the striped actin-rich bulkheads pattern indicative of the BC lumina (Fig. 6 a, Video 7, and Video 8). Conversely, the elongated lumina that still formed always contained the transversal actin stripes. Careful inspection of the live-cell imaging videos (e.g., Video 1 and Video 8) indicated that the disappearance of the transversal bulkheads precedes the formation of a spherical lumen.

**Video 7.** **Formation of a spherical lumen upon Rab35 silencing in vitro*.*** Live-cell time-lapse microscopy showing the growth of a spherical lumen between two differentiating hepatoblasts expressing LifeAct-EGFP upon Rab35 silencing. Images acquired in 10-min intervals. The video displays a 29-h time window at 12,000× normal speed. Scale bar: 10 µm.

**Video 8.** **Formation of a multicellular cyst in vitro upon Rab35 silencing.** Live-cell time-lapse microscopy documenting the formation of a multicellular cyst upon Rab35 silencing in differentiating hepatoblasts expressing LifeAct-EGFP. Images acquired in 10-min intervals. The video displays a 51-h time window at 12,000× normal speed. Scale bar: 10 µm.

To corroborate the loss of the bulkheads in the spherical lumina induced by Rab35 knock-down, we examined their ultra-structure by EM on serial sections and 3D reconstruction of the entire lumen volume. We focused on large cyst-like lumina formed by several cells. Individual EM sections of a cyst-like lumen between five cells showed that the bulkheads that are normally present in the BC lumina were absent (Fig. 6 c). This was confirmed by the 3D model of the lumen based on rendering plasma membranes and TJs (Fig. 6 d). In addition, the TJs between the cells did not protrude into the lumen, as seen at the sagittal cross-section of the 3D model (Fig. 6 d).

### Re-engineering of liver tissue architecture by silencing of Rab35 in vivo

**Figure S3.**
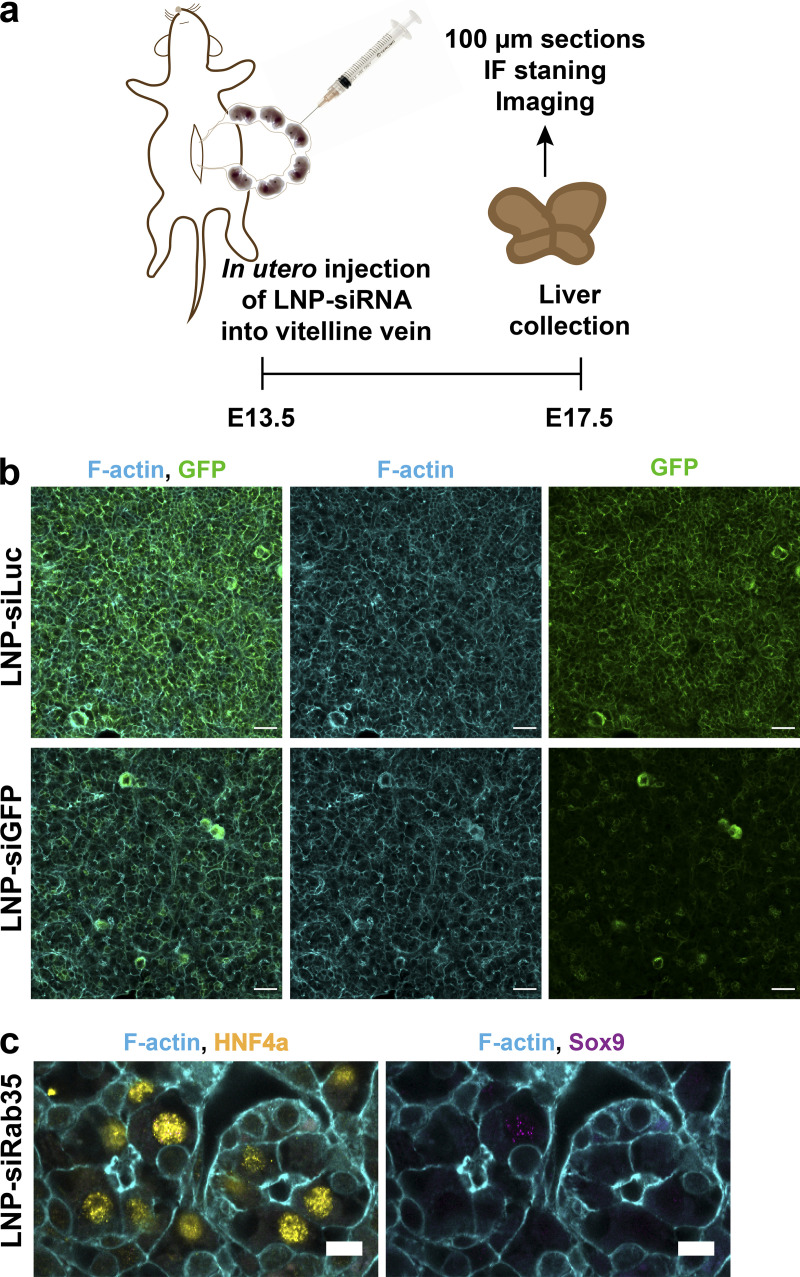
**In utero injection method validated by silencing of GFP in GFP-expressing mice. (a)** Schematic overview of in utero injection experiments. **(b)** Microscopy images showing the down-regulation of GFP signal in GFP-expressing mouse livers via in utero injection of LNP-siGFP in comparison to livers injected with control LNP-siLuc. Scale bar: 30 µm. **(c)** Immunofluorescence (IF) microscopy images of the livers injected with LNP-siRab35 and stained for HNF4a (yellow) and Sox9 (magenta). Scale bar: 30 µm.

If the transversal bulkheads confer to hepatocytes their specific polarity and, consequently, the cell-level anisotropic growth of the apical lumen, one could exploit their loss to reengineer liver tissue, i.e., to predictably modify its structure, particularly the geometric characteristics of the BC network. The structure of liver tissue depends on two types of cell polarity, the polarity of hepatocytes that leads them to form the BC and the vectorial polarity of cholangiocytes that form the bile ducts. Loss of the transversal bulkheads in hepatocytes in vivo should change cell polarity, resulting in a reorganization of cell–cell interactions. If so, the BC should be replaced by bile duct-like epithelial tubes. The complete loss of Rab35 in a knockout mouse line is embryonically lethal (Dickinson et al., 2016), presumably due to cytokinesis defects (Kouranti et al., 2006). To circumvent this problem and deplete Rab35 as in vitro, we took advantage of lipid nanoparticles (LNPs) developed for human therapeutics, enabling the specific delivery of siRNAs to hepatocytes in the liver (Akinc et al., 2010; Zeigerer et al., 2012). To target the E13.5 embryonic liver, we used a method for in utero injection via vitelline vein (Ahn et al., 2018). We first validated the technique on mice expressing membrane-targeted GFP. We performed the in utero injection of LNP-GFP or Luciferase (as control) siRNA in E13.5 embryos and collected the livers after 4 d of development (Fig. S3 a). The GFP signal in the liver was markedly and homogeneously reduced in hepatocytes, whereas different cell types, e.g., hematopoietic cells, were unaffected (Fig. S3 b).

**Figure 7.**
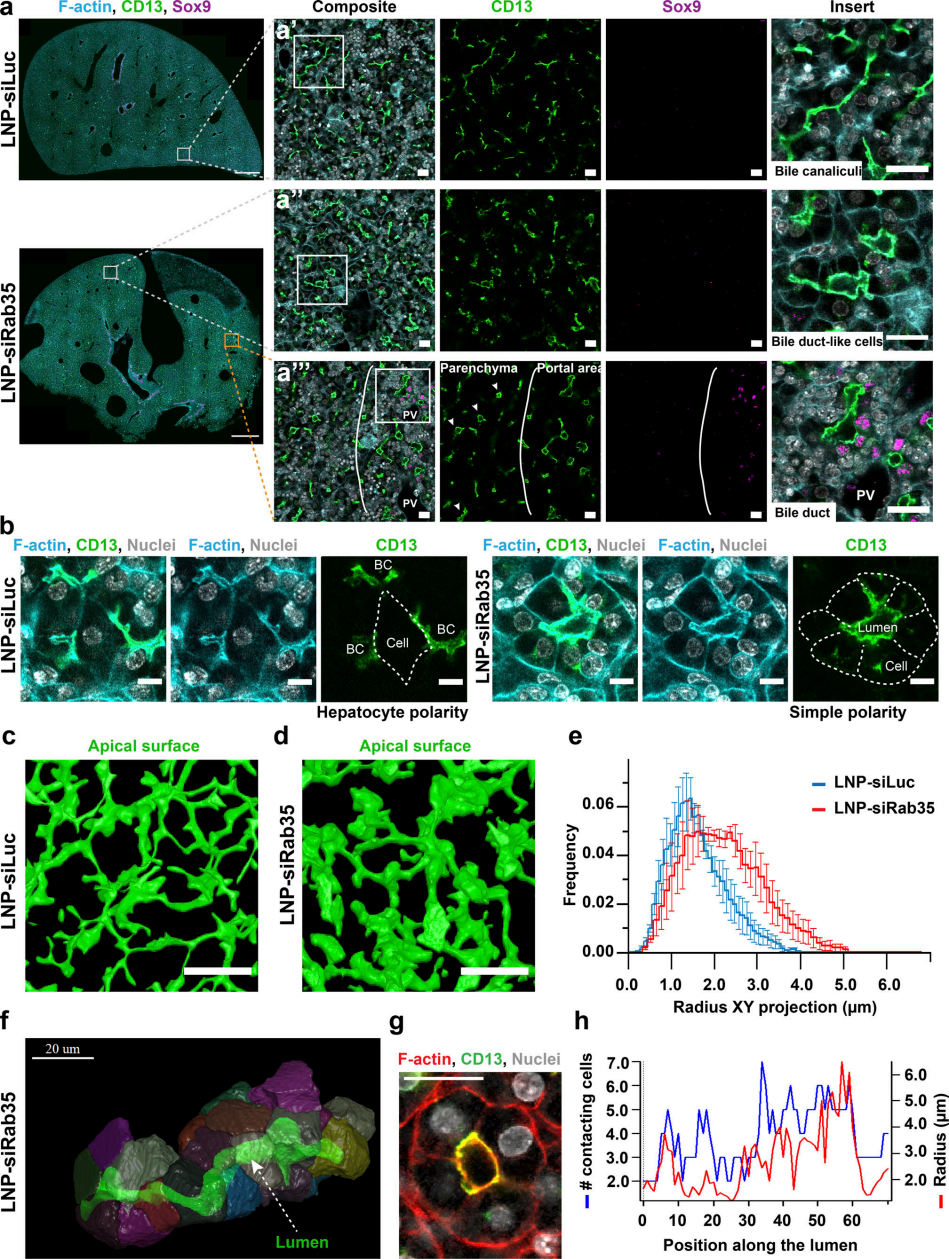
**Silencing of Rab35 in vivo results in altered cell polarity and liver tissue architecture. (a)** Immunofluorescence images of liver tissue collected 4 d after in utero injection of Luciferase (siLuc) and Rab35 (siRab35) siRNAs formulated into LNP via vitelline vein in E13.5 embryos. The square on the low-magnification images (scale bar: 500 µm) shows where the high-resolution image was taken (scale bar: 20 µm). Imaged areas are located in the liver parenchyma, devoid of bile duct cell marker Sox9. The inserts (scale bar: 20 µm) in a’ and a’’ show the difference between BC and bile duct-like lumina in LNP-siRab35 injected liver. Panel a’’’ compares tubular lumina in the parenchyma to the bile duct lumina in the portal area (Sox9-positive cells near portal veins [PV]). **(b)** Immunofluorescence images of liver tissues from panel a show examples of hepatocyte polarity in the control tissue (a single hepatocyte forms multiple lumina per cell) and simple apico-basal polarity in LNP-siRab35 injected liver (cells have a single apical domain oriented toward a shared lumen). **(c and d)** 3D reconstruction of lumina labeled with an apical marker CD13 in 100-µm-thick sections of liver tissue injected with LNP-siLuc (c) and LNP-siRab35 (d). Scale bar: 30 µm. See also Video 9. **(e)** Quantification of the lumen radius distribution based on the 3D reconstructions such as in c and d (*n* = 3, error bars: SEM). **(f)** 3D reconstruction of a tubule in LNP-siRab35–injected livers shows a cylindrical lumen (green) surrounded by multiple cells. See also Video 10. **(g)** A cross-section of the reconstructed tubule in f in the original microscopy image shows organization of the cells around the lumen. Scale bar: 20 µm. **(h)** Quantification of number of cells surrounding the lumen in relation to lumen radius and position along the tubule.

We next formulated the Rab35 siRNA validated in vitro (Fig. 5, d–g) and Luciferase siRNA into LNPs, injected them into embryonic livers, and analyzed the effect using a pipeline of immunostaining, deep tissue imaging and 3D reconstruction (Morales-Navarrete et al., 2015; Fig. 7 a). As in control liver, E17.5 livers injected with LNP-Luciferase siRNA developed normal elongated BC tubules formed by two adjacent hepatocytes (Fig. 7 a’). Strikingly, LNP-Rab35 siRNA injection indeed induced the formation of large tubular structures in the liver parenchyma (Fig. 7 a’’). 3D reconstruction of apical surfaces (marked with CD13) in 100-µm-thick sections revealed the typical appearance of 3D BC network in normal and LNP-Luciferase siRNA-injected livers (Fig. 7 c and Video 9). In contrast, in LNP-Rab35 siRNA-injected livers, the 3D reconstruction showed profound changes in lumen morphology (Fig. 7 d and Video 9). The quantification of the reconstructed lumina showed a general increase in lumen radius (Fig. 7 e), similar to the one observed in vitro (Fig. 5 e). Remarkably, 30% more BC lumina had radii >2 µm compared with control livers (mean ± SEM, control: 26.7% ± 9.4%, siRab35: 57% ± 11.9%).

**Video 9.** **3D reconstruction of luminal network in livers injected with LNP-siLuc or LNP-siRab35.** 3D reconstruction of lumina labeled with an apical marker CD13 in 100-µm-thick sections of liver tissue injected with LNP-siLuc and LNP-siRab35. CD13 staining is shown first, then the 3D reconstruction based on the staining. The veins are shown in red. Scale bar: 30 µm.

The 3D analysis of the tubules suggests that their expanded lumina were not due to a mere dilation of the BC but rather a modification of cell polarity. Reconstruction of segments of large tubular structures revealed that, instead of the characteristic BC lumen formed by two adjacent hepatocytes, here the lumen was formed by four or five conical-shaped cells in each cross-section of the tube, similar to bile ducts (Fig. 7, f–h). Such a reorganization is apparent if one observes the 3D reconstruction of the tubule segment (Video 10), showing the individual cells facing the lumen (Fig. 7, f and g). Importantly, many cells forming the tubes had a single apical surface facing the lumen, as shown in the example (Video 10). Several cells extended the apical surface laterally, connecting it to BC, but without reaching the level of apical surface ramification (biaxial polarity) as in control liver. Such a change in cell polarity is apparent when one compares the vectorial polarity of one of the cells lining the tube with the biaxial polarity of control hepatocytes (Video 10). Consequently, the lumen of the tube is mainly cylindrical with few ramifications, in contrast to the branched BC network in control livers (Fig. 7, b and c).

**Video 10.** **3D reconstruction of a tubule formed in LNP-siRab35-injected liver.** 3D reconstruction of a tubule in LNP-siRab35-injected livers. First, CD13 staining is shown; next, the reconstructed lumen in green and reconstructed cells contributing to the lumen in random colors. The lumen is surrounded by multiple cells. Then, the video zooms on a single cell within the tube with the apical surface highlighted in green. Last, the video shows an example of a cell in control livers and its apical surface (green).

As the tubular structures generated upon Rab35 knock-down are remarkably similar to bile ducts at this developmental stage, we needed to rule out that they may be formed by bile duct cells. First, the tubular structures are present throughout the parenchyma and in proximity to the central vein, i.e., distant from the portal area where the bile ducts are located (Fig. 7 a’’). Second, the cells expressed the hepatocyte marker HNF4a but not the bile duct cell marker Sox9, suggesting that these structures are not mistaken for the bile ducts in the portal area (Fig. 7 a’’’ and Fig. S3 c).

Altogether, these results suggest that loss of Rab35 caused changes in hepatocyte polarity, from biaxial to vectorial, resulting in a reorganization of cell–cell interactions and the reengineering of BC that adopt the morphology of bile duct–like epithelial tubes.

## Discussion

The molecular and physical mechanisms underlying the anisotropy of lumen formation are an emerging area of research. In this study, by searching for a mechanism that could explain the anisotropy of hepatocyte apical lumina, we discovered the existence of specific extensions of the apical membrane sealed by TJs in the lumen between two adjacent hepatocytes. The best analogy we could find for these structures are the bulkheads of boats, ships, and planes. Bulkheads provide structural stability and rigidity, strengthening the structure of elongated vessels. From the physics of thin shells, formation of a tubular lumen with inner pressure and no outlets, such as the forming BC, requires anisotropy of surface tension and/or rigidity of the wall (Landau and Lifshitz, 1986; Berthoumieux et al., 2014). The apical bulkheads are structural elements that can provide such anisotropy and mechanical stability to the elongating cylindrical lumen under inner pressure. Interestingly, they follow a quasi-periodic pattern, whose distance is in the range of the diameter of the lumen, as in human-made constructions, where bulkheads are load-bearing structures. Here, they provide forces required for maintaining a nonspherical lumen. One can consider the cylinder with bulkheads as a “chain of spheres,” which is mechanically stable. The bulkheads in ships can also act as (semi)watertight compartments to prevent seeping of water to other parts of the ship. Similarly in the BC, they may act as valves ensuring directionality of bile flux in a nonperistaltic contractility. Additionally, the bulkheads may serve as hot-spots of contractility to facilitate bile flux, as shown in vivo (Watanabe et al., 1991; Meyer et al., 2017). Mechanistically, the position of the bulkheads could be determined by mechano-sensing mechanisms coupled to the tension and local curvature through the actin cortical mesh (Meyer et al., 2020). The elongation of the apical lumen also entails the movement and rearrangement of cell–cell contacts, which are accompanied by the formation of new bulkheads (Fig. 1 d and Fig. 2). Upon loss of the bulkheads caused by Rab35 down-regulation, the apical surfaces of hepatocytes lose their anisotropic growth, and the elongated lumina convert into spherical. Remarkably, we succeeded in reengineering liver tissue structure by down-regulation of Rab35 in vivo*.* This resulted in the modification of the cell polarity of hepatocytes, which, instead of forming BC, self-organized into tubular epithelial structures resembling bile ducts. It will be interesting to assess whether such morphological changes have consequences on hepatocyte cell fate and function.

We showed that the apical bulkheads are present in embryonic and adult liver, suggesting that they are not a cell culture artifact but have physiological relevance. In addition, the elongation assisted by apical bulkheads does not rely on cell division and therefore can explain the BC extension in quiescent differentiated hepatocytes in later stages of liver development (Yang et al., 2017). Their dynamic and adaptable nature fit the requirements of a growing, branching, and fusing BC network in vivo. To our knowledge, these structures were never described before despite several ultrastructural studies of liver from different species. They were probably not observed by EM before or mistaken for folds and ramifications due to the complexity of the BC network in the liver, or interpreted as septa in 2D EM sections of adult hepatocytes (Kawahara and French, 1990). Their visualization requires a 3D EM reconstruction.

We obtained several cues to the mechanisms underlying the apical bulkheads formation from the morphological analysis and functional screen by RNAi. First, the bulkheads are characterized by a T-shaped arrangement of TJs, which seals the two halves of the bulkheads (Fig. 1 b and Fig. 3 f). To our knowledge, this organization is unprecedented in polarized cells. Second, given that the TJs are connected to actin filaments, it is no surprise that the bulkheads contain F-actin transversally to the lumen elongation, thus introducing anisotropy in apical surface tension. Third, by a focused RNAi screen for established regulators of cell polarity, we found that the small GTPase Rab35 is required for the formation of the apical bulkheads and hepatocyte lumen shape. Based on previous work (Kouranti et al., 2006; Klinkert and Echard, 2016; Dambournet et al., 2011; Bhat et al., 2020; Zhang et al., 2009; Chevallier et al., 2009; Salvatore et al., 2018; Allaire et al., 2013; Marat et al., 2012; Jewett et al., 2017; Egami et al., 2011; Klinkert et al., 2016), Rab35 may contribute to the formation of these structures directly or indirectly, and we envision the following nonexclusive possibilities. Rab35 is a regulator of endosomal recycling (Kouranti et al., 2006; Klinkert et al., 2016; Mrozowska and Fukuda, 2016) and may control the intracellular distribution and function of apical recycling endosomes to deliver transmembrane proteins, e.g., junction components, at the site of bulkheads initiation and/or growth. The T arrangement of the TJs could originate from the junctions longitudinal along the tubule (horizontal bar in the T), and Rab35 may support the zip-up along the ridgeline (vertical bar of the T), either from the bottom or from the top of the tube. However, in addition to protein transport, generation of the apical bulkheads may require the formation of a mechanical support, either by delivering molecules to specific areas of the apical surface or by the apical vesicles anchored to the cytoskeleton (actin and microtubules) to project force into the apical bulkheads. The presence of clusters of vesicles at the base of the bulkheads as visualized by EM supports this view. Preliminary results suggest that Rab35 indeed localizes to sub-apical vesicles (Bebelman and Zerial, unpublished data). In addition, Rab35 is also known to coordinate membrane trafficking with the organization of the actin cytoskeleton (Klinkert and Echard, 2016; Chua et al., 2010). It may regulate actin remodelling to form the F-actin of the bulkheads, similar to its function in promoting the formation of F-actin–rich tunneling nanotubes in neuronal cells (Bhat et al., 2020). In the context of the apical bulkheads, it would orient the filaments between the TJs and the vesicles at the base, providing the aforementioned mechanical function. Rab35 could regulate the local phosphoinositide content via, e.g., inositol polyphosphate 5-phosphatase OCRL, nucleation and/or dynamics of the F-actin at the bulkheads, e.g., via MICAL1 or unknown hepatocyte-specific effectors (Chaineau et al., 2013; Dambournet et al., 2011; Frémont et al., 2017). Alternatively, Rab35 could play an indirect role by modulating signaling pathways, e.g., integrin-based cell adhesion (Allaire et al., 2013) and/or gene expression. Also, the function of genes implicated in hepatocyte polarity, e.g., *Par1b*, *Pard3*, *Cldn2*, *Cldn3*, and *Lkb1* (cAMP-Epac-MEK-AMPK pathway regulating BC network formation) should be revisited specifically in the context of the bulkheads and anisotropy of lumen elongation (Wang et al., 2014; Fu et al., 2010; Son et al., 2009; Grosse et al., 2013; Slim et al., 2013; Homolya et al., 2014; Fu et al., 2011; Woods et al., 2011).

Our data thus suggest that transversal mechanical coupling between hepatocyte apical surfaces underlies the formation of BC and provide new insights into the longstanding problem of lumen morphogenesis in embryonic liver.

## Materials and methods

### Animals and animal handling

Animal experiments were conducted in accordance with German animal welfare legislation in pathogen-free conditions in the animal facility of the Max Planck Institute of Molecular Cell Biology and Genetics (MPI-CBG), Dresden, Germany. Mice were maintained in a conventional barrier animal facility with a climate-controlled environment on a 12-h light/12-h dark cycle, fed ad libitum with regular rodent chow. Protocols were approved by the Institutional Animal Welfare Officer (Tierschutzbeauftragter), and necessary licenses were obtained from the regional Ethical Commission for Animal Experimentation of Dresden, Germany (Tierversuchskommission, Landesdirektion Dresden). For primary hepatoblast isolations, embryonic livers were collected from timed-pregnant (E13.5–E14.5) wild-type mice C57BL/6JOlaHsd (Harlan Laboratories/Envigo) or C57BL6/JRj (Janvier Labs), or transgenic lines LifeAct-EGFP (Riedl et al., 2010), ROSAmT/mG (Muzumdar et al., 2007), or the in-cross of the two transgenic lines. For in utero LNP injection experiments, the GFP-expressing embryos were generated by crossing of ROSAmT/mG females with PGKCre(J) males (Lallemand et al., 1998). The transgenic or wild-type embryos were injected in utero via the vitelline vein at E13.5 and livers collected at E16.5–E17.5.

### Dlk1^+^ hepatoblast isolation

Hepatoblasts were isolated as a Dlk1^+^ fraction using magnetic cell separation, according to a modified published protocol (Tanimizu et al., 2003). Timed-pregnant mice (E13.5–14.5) were sacrificed by cervical dislocation. 16–24 embryonic livers were collected, fragmented, and incubated in liver perfusion media (Thermo Fisher Scientific; cat. no. 17701–038) for 20 min in a 37°C water bath. The liver pieces were digested in Liver Digest Medium (Thermo Fisher Scientific; cat. no. 17703–034,) supplemented with 10 µg/ml DNase I (Sigma-Aldrich; cat. no. DN25) for a further 20 min. Erythrocytes were lysed in red blood cell lysis buffer (155 mM NH_4_Cl, 10 mM KHCO_3_, and 0.1 mM Na_4_ EDTA, pH 7.4). Digested cells were incubated with blocking antibody Rat Anti-Mouse CD16/CD32 (BD Biosciences; cat. no. 553142; 1:100) for 10 min, then with Anti-Dlk mAb-FITC (MBL; cat. no. D187-4; 1:40) for a further 15 min. After washing with a buffer (0.5% BSA, and 2 mM EDTA in PBS), cells were incubated with Anti-FITC MicroBeads (Miltenyi Biotec; cat. no. 130–048-701; 1:10) for 15 min and separated on a magnetic column (Miltenyi Biotec; cat. no. 130–024-201) according to the manufacturer’s protocol.

### Hepatoblasts culture and differentiation

Culture wells were precoated either with 10 µg/ml fibronectin (Sigma-Aldrich; cat. no. F1141) in PBS or with 10 vol/vol % Matrigel (BD Biosciences; cat. no. 356231) in ice-cold PBS for at least 30 min at 37°C. Two culture protocols were used in the study. In protocol 1, which was used for the RNA sequencing (RNA-seq) experiment, isolated cells were diluted in differentiation media (MCDB131, no glutamine [GIBCO BRL; cat. no. 10372019], 5% FBS, 2 mM L-glutamine [Thermo Fisher Scientific; cat. no. M11-004], 1× ITS-X [GIBCO BRL; cat. no. 51500–056], and 0.1 µM dexamethasone [Sigma-Aldrich; cat. no D1756-25MG]) containing 4% Matrigel and seeded on fibronectin-coated plates.

In protocol 2, Dlk1^+^-enriched cells were seeded on Matrigel-coated plates in expansion media (DMEM/F-12, GlutaMAX supplement [Thermo Fisher Scientific; cat. no. 31331028], 10% FBS, 1× ITS-X [GIBCO BRL; cat. no. 51500–056], 0.1 µM dexamethasone [Sigma-Aldrich; cat. no D1756-25MG], 10 mM nicotinamide [Sigma-Aldrich; cat. no. N0636-100G], 10 ng/ml human HGF [in-house production], and 10 ng/ml mouse EGF [in-house production]). 24 h later, the cells were overlaid with differentiation media containing Matrigel to the final 5%. In 96-well plates, cells were seeded at the density 13,000 cells/well in 24-well plates at the density 60,000 cells/well. Cells were cultured for 5 d at 37°C, 5% CO_2_, with one additional differentiation media change. Dlk1^+^ cells from E14.5 livers contained ∼10% cells positive for bile duct cell marker Sox9 and were used in the experiments to optimize the growth of bile duct cysts. For other experiments, Dlk1^+^ cells from E13.5 livers were used, as all the cells gave rise to hepatocytes with BC in the protocol 2 culture conditions.

### Primary hepatocytes

Primary hepatocytes were isolated from male 8–12-wk-old mice according to the well-established collagenase perfusion protocol (Klingmüller et al., 2006). They were lysed immediately for RNA isolation or cultured in a collagen sandwich in 24-well plates (200,000 cells/well) in William’s E medium (Pan Biotech; cat. no. P04-29150) supplemented with 10% FBS, 100 nM dexamethasone (Sigma-Aldrich; cat. no D1756-25MG), and penicillin/streptomycin until they polarized (Zeigerer et al., 2017). The polarized hepatocytes were fixed with 4% PFA for 30 min.

### Live-cell time-lapse microscopy

For the live-cell video microscopy, LifeAct-EGFP (Riedl et al., 2010) and ROSAmT/mG (Muzumdar et al., 2007) mouse strains were crossed, and EGFP^+^ embryos were collected for the Dlk1^+^ cells’ isolation. The Dlk1^+^ cells were plated (transfected with siRNA) and imaged from day 3 of the culture in the differentiation media on an epifluorescent microscope Zeiss Axiovert 200 M with an incubator (37°C, 5% CO_2_) using an 20× objective (NA 0.5) in 10-min intervals for ∼52 h. To image the localization of EGFP-Rab35, the Dlk1^+^ cells were isolated from ROSAmT/mG embryos and transduced with a recombinant adenovirus (AdenoEGFP-Rab35) at day 2 of the culture. The cells were imaged on day 3 in 5-min intervals for up to 24 h.

### Immunofluorescence staining and confocal imaging

Cultured cells were fixed with 3% PFA for 15 min at RT, washed 3× with PBS, permeabilized with 0.1% Triton X-100 in PBS for 5 min at RT, and blocked with 0.5% FBS in PBS for min 30 min at RT. Primary antibodies were diluted in the blocking solution, rat monoclonal anti-CD13 (Novus; cat. no. NB100-64843; RRID:AB_959651; 1:500), rabbit polyclonal anti-ZO-1 (Thermo Fisher Scientific; cat. no. 40–2200; RRID:AB_2533456; 1:200), goat polyclonal anti-mouse podocalyxin (R&D Systems; cat. no. AF1556-SP; RRID:AB_354858; 1:400), rat monoclonal anti-integrin β1 (Millipore; cat. no. MAB1997; RRID:AB_2128202; 1:200), rabbit monoclonal anti–E-cadherin (Cell Signaling Technology; cat. no. 3195; RRID:AB_2291471; 1:200), rat monoclonal anti-mouse CD326 EpCAM-FITC, clone G8.8 (eBioscience; cat. no. 11–5791-82; 1:100) and goat polyclonal anti-Sox9 (R&D Systems; cat. no. AF3075; RRID:AB_2194160; 1:200) and were incubated 1 h at RT or overnight at 4°C. Secondary antibodies (and/or phalloidin-Alexa dyes [Thermo Fisher Scientific; 1:250] and DAPI [1 mg/ml; 1:1,000]) were incubated for 1 h at RT. For staining with rabbit polyclonal anti-Rab35 (Antibody Facility MPI-CBG Dresden; H26952; 1:1,000), the cells were permeabilized with 0.05% saponin and blocked with 3% BSA in PBS instead. Finally, cells were washed with PBS and imaged in the culture plates on inverted laser scanning confocal microscopes Olympus Fluoview 1000 (objectives 40×/0.9/air, 60×/1.2/water) or Zeiss LSM 700 (objectives 40×/1.2/water, 20×/0.8/air).

### SMLM

SMLM experiments were performed on a Nikon Eclipse Ti microscope, using a 100×/1.49 NA oil immersion objective together with a 1.5× postmagnification lens (Franke et al., 2019). All measurements were performed with an active perfect focus control. Prior to acquisition, samples were irradiated in epifluorescence illumination mode to turn emitters, which were out of focus in the acquisition HILO illumination scheme, into the dark state. The length of the acquisition was set to capture the majority of emitters, i.e., imaging was concluded when only a very minor number of active emitters was detectable. When a critically low spot density was first reached, an acquisition scheme of 1 frame with low 405-nm excitation (activation) followed by 5 consecutive frames with 641-nm excitation was used. Typical acquisition lengths were 60,000–200,000 frames with 20-ms integration time and 641-nm excitation. Raw image stacks were analyzed with rapidSTORM 3.2 (Wolter et al., 2012). The full-width-at-half-maximum (FWHM) was set as a free fit parameter, but in the limits of 275–650 nm, which corresponds to an axial range of ∼1 µm (Franke et al., 2017), the fit window radius was set to 1,200 nm and the intensity threshold to 1,000 photons, while all other fit parameters were kept from the default settings in rapidSTORM 3.2. Linear lateral drift correction was applied by spatio-temporally aligning distinct structures to themselves. This was facilitated by color-coding of the temporal coordinate with the built-in tool.

### Transmission EM

In vitro cultures of hepatoblasts grown in 24-well plates were fixed by adding warm 2% glutaraldehyde in 200 mM Hepes, pH 7.4, to the culture medium at a 1:1 ratio and incubated for 5 min at 37°C. Then the fixative and medium mixture was replaced by adding fresh 1% glutaraldehyde in 200 mM Hepes, pH 7.4, and samples incubated at 37°C for another 2 h, then at RT overnight. For resin embedding, samples were post-fixed with 1% osmium tetroxide and 1.5% potassium ferricyanide for 1 h on ice, then contrasted en bloc with 2% aqueous uranyl acetate for 2 h at RT, dehydrated with a graded ethanol series, 70–80–90–96%, each for 10 min, and 4× 100%, each for 15 min, progressively infiltrated with LX-112 epoxy resin (Ladd Research Industries) and eventually polymerized at 60°C for 2 d. The plastic of the plate was broken off to release resin disks with a cell monolayer on one side. Disks were cut into small pieces that were remounted for longitudinal sectioning.

To collect the mouse embryonic liver, a pregnant mouse was sacrificed, and livers were dissected from embryos and cut into a few pieces, which were immersion-fixed with 4% paraformaldehyde in 200 mM Hepes, pH 7.4, and 1 mM CaCl_2_ overnight. To fix the adult liver, mice were transcardially perfused with 4% PFA in PBS for 15 min and post-fixed overnight. Before resin embedding, liver tissue was cut in small pieces and additionally fixed with 1% glutaraldehyde in 200 mM Hepes, pH 7.4. Tissue was processed as described above except that EPON resin was used for embedding. Tissue was sectioned at random orientation.

Serial, 90-nm-thin sections were cut using a Leica Ultracut UCT ultramicrotome and deposited on formvar-coated, slot, copper grids. Sections were contrasted with 0.4% lead citrate for 1 min and imaged in a Tecnai T12 transmission electron microscope (Thermo Fisher Scientific), operated at 100 kV and equipped with an axial 2k CCD camera (TVIPS).

Z-stack of images of serial sections were aligned using a TrackEM2 plugin in Fiji (Cardona et al., 2012). The liver apical membrane, bile canaliculus lumen, and junctional complex were segmented on aligned image stacks using IMOD (Kremer et al., 1996) in order to reconstruct a 3D model in IMOD or Blender (Blender Online Community, 2018).

### siRNA design, synthesis, and transfection

Design of siRNA was performed using in-house software, first by testing all available sequences on the specificity for the target in mouse transcriptome (RefSeq in Pubmed), followed by elimination of sequences with significant complementarity to mouse miRNA, GC content <25% and >75%, and immune responsive ones (like UGU, UGUGU, etc.). In addition, sequences were filtered using Reynolds rules (Reynolds et al., 2004). Six siRNAs with highest functionality score were selected (Table S2) and synthesized by the solid-phase phosphoramidite method, purified by ion-exchange HPLC, and verified by liquid chromatography–mass spectrometry (Farzan et al., 2017). Pyrimidines in the sense strand and before A in antisense strand (UA, CA dinucleotides) were 2’-O-methylated (shown by lowercase letters in the sequence), and both strands were 3′-modified with phosphorothioate dithymidylate to enhance nuclease stability. Working stocks were prepared by diluting siRNAs to 10 µM in 10 mM Tris-HCl, pH 7.5. siRNAs were transfected using transfection reagent Lipofectamine RNAiMAX (Thermo Fisher Scientific; cat. no. 13778075) according to the reverse transfection protocol provided by the manufacturer. The final concentration per well was 10 nM siRNA and 0.1 vol/vol % Lipofectamine RNAiMAX. Control luciferase and GFP siRNA were previously published: control siRNA luciferase (Zeigerer et al., 2012; sense 5′-cuuAcGcuGAGuAcuucGAdTsdT-3′, antisense 5′-UCGAAGuACUcAGCGuAAGdTsdT-3′), and GFP siRNA (Gilleron et al., 2013; sense 5′-ACA​UGA​AGC​AGC​ACG​ACU​UTT-3′, antisense 5′-AAG​UCG​UGC​UGC​UUC​AUG​UTT-3′).

### Protein extraction and Western blotting

Cultured cells were lysed for 20 min in ice-cold SDS lysis buffer (20 mM Tris-HCl, pH 7.5, 150 mM NaCl, 1 mM EDTA, 1 mM EGTA, 1% SDS, 1% NP-40 [IGEPAL CA-630], and freshly added 1/1,000 CLAAAP [chymostatin, leupeptin, antipain, aprotinin, APMSF [(p-Amidinophenyl)methanesulfonyl fluoride], and pepstatin), and 1/100 Phosphatase Inhibitor Cocktail 2 and 3 (Sigma-Aldrich). Per condition, 5 wells of a 96-well plate were pooled together into a total of 125 µl of the SDS lysis buffer. The lysates were sonicated for 3 min and spun at 13,000 × *g* for 10 min, 4°C. Protein concentration was measured with DC Protein Assay (Bio-Rad; cat. no. 500–0116). The samples were separated on 15% SDS-PAGE and transferred onto nitrocellulose membrane. Membranes were blocked and incubated with primary antibodies rabbit polyclonal anti-Rab35 (Antibody Facility MPI-CBG Dresden; F18256; 1:1,000) and mouse monoclonal anti–γ-tubulin (clone GTU-88; Sigma-Aldrich; cat. no. T6557; RRID:AB_477584; 1:2,000) and secondary HPR-conjugated antibodies (1:10,000) in 5% dry milk, 10 mM Tris-HCl, pH 8.0, 200 mM NaCl, and 0.1% Tween20. The bound antibody was detected with the ECL Western Blotting detection kit (GE Healthcare; cat. no. RPN2209) on Hyperfilm ECL (Amersham GE Healthcare). The quantification of Western blots was done with Image J (Miller, 2010). Statistics were calculated and plots were generated in R (R Core Team, 2019).

### RNA isolation and RT–quantitative PCR (qPCR)

Total RNA was isolated using the RNeasy Mini Kit (Qiagen; cat. no. 74104 50) including the DNase I (Qiagen; cat. no. 79254) treatment step. Cells were lysed with provided RNeasy lysis buffer supplemented with DTT. cDNA was synthesized using the ProtoScriptII First Strand cDNA Synthesis Kit (NEB; cat. no. E6560S), following the manufacturer’s protocol with the Random Primer Mix and the RNA denaturation step. qPCR was performed on Roche LightCycler 96 in 10-µl reactions using FastStart Essential DNA Green Master (Roche; cat. no. 06402712001). A housekeeping gene Rplp0 was used as an endogenous reference gene. The qPCR primers for Rplp0 were forward, 5′-AGA​TTC​GGG​ATA​TGC​TGT​TGG​C-3′; and reverse, 5′-TCG​GGT​CCT​AGA​CCA​GTG​TTC-3′. The qPCR primers for Rab35 were forward, 5′-TGT​CAA​CGT​CAA​GCG​ATG​G-3′; and reverse, 5′-GGT​CAT​CAT​TCT​TAT​TGC​CCA​CT-3′. Normalized relative gene expression value and percent knock-down was calculated using the ΔΔCq method (Haimes and Kelley, 2010). Statistics were calculated and plots were generated in R (R Core Team, 2019).

### Bulk RNA-seq

The following samples were collected in four biological replicates: E14.5 Dlk1^+^ hepatoblasts isolated and immediately processed for RNA isolation, in vitro differentiated hepatocytes from E14.5 Dlk1^+^ hepatoblasts differentiated according to the culture protocol 1, and mature hepatocytes isolated from adult male mice following published protocols (Klingmüller et al., 2006) and immediately processed for RNA isolation. The integrity of RNA was measured by an Agilent 2100 Bioanalyzer. Preferentially, only samples with the RNA integrity number >9.0 were used. 1 µg mRNA was isolated from the total RNA by poly-dT enrichment using the NEBNext Poly(A) mRNA Magnetic Isolation Module according to the manufacturer’s instructions. Final elution was done in 15 μl 2× first-strand cDNA synthesis buffer (NEB; NEBNext). After chemical fragmentation by incubating for 15 min at 94°C, the sample was directly subjected to the workflow for strand-specific RNA-seq library preparation (NEBNext Ultra RNA Library Prep Kit for Illumina). For ligation, custom adaptors were used (Adaptor-Oligo 1: 5′-ACA​CTC​TTT​CCC​TAC​ACG​ACG​CTC​TTC​CGA​TCT-3′, Adaptor-Oligo 2: 5′-P-GATCGGAAGAGCACACGTCTGAACTCCAGTCAC-3′). After ligation, adapters were depleted by an XP bead purification (Beckman Coulter) adding bead in a ratio of 1:1. Indexing was done during the following PCR enrichment (15 cycles, 65°C) using custom amplification primers carrying the index sequence indicated with “NNNNNN” (Primer1: Oligo_Seq 5′-AAT​GAT​ACG​GCG​ACC​ACC​GAG​ATC​TAC​ACT​CTT​TCC​CTA​CAC​GAC​GCT​C​TTCCG​ATC​T-3′; primer2: 5′-GTG​ACT​GGA​GTT​CAG​ACG​TGT​GCT​CTT​CCG​ATC​T-3′; primer3: 5′-CAA​GCA​GAA​GAC​GGC​ATA​CGAG​AT NNNNNN GTGACTGGAGTT-3′). After two more XP bead purifications (1:1), libraries were quantified using the Qubit dsDNA HS Assay Kit (Invitrogen). For Illumina flowcell production, samples were equimolarly pooled and distributed on all lanes used for 75-bp single-read sequencing on an Illumina HiSeq 2500, resulting in, on average 30 Mio-sequenced fragments per sample.

### Recombinant adenovirus production and rescue experiments

Recombinant adenovirus to express EGFP-fused Rab35 (human RAB35 cDNA, transcript variant 1 [NM_006861.7]) was produced using the AdEasy Vector System (Qbiogene) developed by He et al. (1998). A linker GGGGSGGGGS was introduced between EGFP and RAB35. The RAB35 fragment with the linker extension was amplified from the Addgene plasmid #47424, a gift from Peter McPherson, (McGill University, Montreal, Canada; Allaire et al., 2010), and subcloned into pEGFP-C3 vector (Clontech) using ScaI and BamHI restriction sites (Rab35-ScaI-2GGGGS-F: 5′-GAG​AAG​TAC​TAC​ggc​ggc​ggc​ggc​agc​ggc​ggc​ggc​ggc​agc​ATG​GCC​CGG​GAC​TAC​GAC​CA-3′, Rab35-BamHI-R: 5′-GAG​AGG​ATC​CTC​ATT​AGC​AGC​AGC​GTT​TCT​TTC​G-3′).

The EGFP-linker-RAB35 fragment was cloned into a transfer vector pShutle-CMV (AdEasy Vector System, Qbiogene) using SalI and HindIII restriction sites (EGFP-SalI-F: 5′-ATC​TGG​TAC​CGT​CGA​CAT​GGT​GAG​CAA​GGG​CGA​GGA​G-3′, Rab35-HindIII-R: 5′-TCT​TAT​CTA​GAA​GCT​TTT​AGC​AGC​AGC​GTT​TCT​TTC​GTT​TAC-3′).

The recombinant transfer vector was linearized by PmeI and transformed into electro-competent *Escherichia coli* strain BJ5183-AD-1 (Stratagene; cat. no. 200157–11) for in vivo recombination with pAdEasy vector. A positive clone was amplified in *E. coli* DH5α and linearized with PacI prior the transfection into the packaging cell line QBI-293A (Qbiogene HEK-293A cell derivative cultured in DMEM High Glucose [Gibco; cat. no. 41966–029] with 5% FBS [heat inactivated]). Virus was amplified and purified via OptiPrep-gradient (iodixanol 60 wt/vol% solution; Axis Shield; cat. no. 1114542). The control EGFP-only virus was produced similarly.

E13.5 Dlk1^+^ hepatoblasts were seeded and transfected as described above with Luc siRNA or Rab35 siRNA #4. 72 h later, the cells were infected with the recombinant adenovirus (EGFP or EGFP-Rab35) at dilutions 1:1,000 and 1:100, respectively. The cells were cultured for two more days, fixed, and stained with phalloidin–Alexa 647 and DAPI. From the acquired images, the rescue of the lumen phenotype was quantified.

### In utero siRNA-LNP injection

For use in vivo, siRNA oligos were formulated into LNPs with C12-200 lipoid as previously described (Love et al., 2010). siRNA-LNPs were delivered in utero into E13.5 embryonic livers via vitelline vein as described elsewhere (Ahn et al., 2018). We optimized the concentration of siRNA-LNPs to 5 mg/kg body weight and the length of the treatment to 4 d using siRNAs-LNPs targeting GFP mRNA (Gilleron et al., 2013) in ROSAmG embryos (generated from the cross of ROSA mG/mT × PGKCre[J] lines). The weight of the embryos was estimated based on the published results (Kulandavelu et al., 2006). Briefly, the pregnant mice were anesthetized in a narcosis box with isoflurane at 5% then placed on a heated stage attached to a narcosis mask flowing isoflurane at 2–3%. Analgesia was ensured by injecting 4 mg/kg of metamizol right before surgery and maintained by adding 1.33 mg/ml of the same drug in the drinking water until sacrifice. The abdomen of the mouse was shaved and then sterilized with ethanol; the eyes were protected from desiccation using hydration cream. The uterus was exposed via vertical laparotomy. The embryos were then injected with 5 µl of LNPs at 5 mg/kg. The success of the injection was assessed by blood clearance from the targeted vessel. Embryos of the same mother were randomly assigned to be noninjected, injected with control siRNA, or injected with the targeting siRNA. The injections were performed using pulled needles from manually labeled glass capillaries. After injections, embryos were placed back in the abdomen, and the peritoneal cavity was closed by suturing. The epidermis was then closed with surgical clips. At the end of the surgery, the mice were placed close to a heating lamp and monitored until complete awakening. The livers were collected at E17.5.

### Liver tissue staining with optical clearing

Embryonic livers were fixed by PFA immersion (4% PFA, 0.1% Tween20, and PBS) for 2 h at RT and overnight at 4°C. The PFA was neutralized by overnight incubation in 50 mM NH_4_Cl in PBS. The livers were later stored in PBS at 4°C until processing. The livers were mounted in 4% low-melting agarose in PBS and cut into 100-µm-thick sections on a vibratome (Leica VT1200S). For deep tissue imaging, tissue sections were permeabilized with by 0.5% Triton X-100 in PBS for 1 h at RT. The primary antibodies rat monoclonal anti-CD13 (Novus; NB100-64843; RRID:AB_959651; 1:500) and rabbit monoclonal anti-Sox9 (clone EPR14335-78; Abcam; cat. no. ab185966; RRID:AB_2728660; 1:500) were diluted in Tx buffer (0.2% gelatin, 300 mM NaCl, and 0.3% Triton X-100 in PBS) and incubated for 2 d at RT. After washing 5 × 15 min with 0.3% Triton X-100 in PBS, the sections were incubated with secondary antibodies donkey anti-rat 568 (BIOTIUM; cat. no. 20092; 1:1,000), donkey anti-rabbit 647 (Thermo Fisher Scientific; cat. no. A31573; 1:1,000), and DAPI (1 mg/ml; 1:1,000) and phalloidin–Alexa 488 (Thermo Fisher Scientific; cat. no. A12379; 1:150) for another 2 d. After washing 5 × 15 min with 0.3% Triton X-100 in PBS and 3 × 1 min with PBS, the optical clearing started by incubating the slices in 25% fructose for 4 h, continued in 50% fructose for 4 h, 75% fructose overnight, 100% fructose (100% wt/vol fructose, 0.5% 1-thioglycerol, and 0.1 M phosphate buffer, pH 7.5) for 6 h, and finally overnight in SeeDB solution (Ke et al., 2013; 80.2% wt/wt fructose, 0.5% 1-thioglycerol, and 0.1 M phosphate buffer). The samples were mounted in SeeDB.

### Quantification and statistical analysis

#### 3D reconstruction of BC

Optically cleared 100-µm liver sections were imaged with an upright multiphoton laser-scanning microscope (Zeiss LSM 780 NLO) equipped with gallium arsenide phosphide detectors. Liver slices were imaged twice at low (20×/0.8 Zeiss objective) and high resolution (63×/1.3 Zeiss objective; 0.3 µm voxel size), respectively. Low-resolution overviews of the complete liver sections were created and used to select for regions where enlarged apical membranes were apparent. Selected regions (∼300 µm × 300 µm × 100 µm; x, y, z) were then acquired at high resolution. High-resolution images were processed and BC-segmented, based on CD13 staining, with the Motion Tracking software as described (Morales-Navarrete et al., 2015; Morales-Navarrete et al., 2016). Local lumen radius distribution was calculated by assuming a maximal radius of 10 µm.

For cells segmentation, a selected region of an image (∼70 µm × 70 µm × 60 µm; x, y, z) was denoised using the PURE-LET method (Luisier et al., 2010), i.e., through the “PureDenoise” plugin in ImageJ, with Cycle-spin = 10 and Multiframe = 11. Shading and uneven illumination were then corrected using BaSiC algorithm (Peng et al., 2017) and Rolling Ball Background Subtraction plugins in Fiji, respectively. The preprocessed image was imported to Motion Tracking, and apical membranes were reconstructed as above. Cells surrounding an apical tube were segmented using the 3D active mesh approach with phalloidin staining as a marker of cell borders (Morales-Navarrete et al., 2015).

#### Lumen radius quantification

To quantify the effect of Rab35 silencing and Rab35 rescue on lumen morphology in vitro, a custom script was written for Fiji to segment lumina on microscopy images based on the actin signal (phalloidin–Alexa 647) and extract region statistics. For the rescue experiment, the segmentation mask was set so that only lumina with a minimum (70%) overlap with GFP channel (expressed protein) were kept for the analysis (the cells that actually express the protein). The script contained a pause for segmentation verification and manual correction. For quantifying lumen radius, we used “local thickness” as descriptor, which can be computed with a Fiji plugin (https://imagej.net/Local_Thickness). The local thickness at any interior point of an object is defined as the diameter of the largest circle that contains the point and completely fits into the object. For each lumen, the local thickness histogram, as well as the average local thickness, was computed. Then, the local thickness histogram of each object was normalized. To account for the different size of the objects, each normalized histogram was multiplied by a weighting factor $w_{i}$, which is proportional to the estimated volume of the object i. Without losing generality, we defined w $w_{i}=\;A_{i}^{3/2}$, where $A_{i}\;$ is the number of pixels belonging to the object. Then, the histograms of all the objects in each image were summed up and normalized (i.e., to discard the effect of differences in the total amount of apical membrane between images). Finally, the averaged histograms (first over different images and then between different experiments *n* = 3) are reported. Error bars show the SEM per bin. The histogram quantification was performed using MATLAB R2020b.

#### Gene expression analysis

Basic quality control of raw sequencing data was performed with FastQC v0.11.2 (Andrews, 2020). Reads were mapped to the mouse genome reference assembly GRCm38, and genes of the Ensembl release v92 (Zerbino et al., 2018) were quantified using STAR v2.5.2b (Dobin et al., 2013). The read duplication level was assessed using MarkDuplicates from Picard tools v2.10.2 (Broad Institute, 2018) and dupRadar v1.8.0 (Sayols et al., 2016). The count data of the samples were filtered for genes with >10 counts in any of the samples and served as input for DESeq2 v1.22.2 (Love et al., 2014) to identify differentially expressed genes using a log2fold-change threshold of 1 and an adjusted P-value cut-off of 0.01. The heatmap was generated using R package gplots (function heatmap.2).

### Online supplemental material

Fig. S1 shows that the hepatoblast culture system also supports the growth and polarization of primary bile duct cells. Fig. S2 provides additional data on selected polarity-related candidates of the siRNA screen. Fig. S3 shows a scheme and validation of the in utero injection method to deliver LNP-siRNAs into embryonic livers using a GFP-expressing mouse line. Video 1, Video 2, Video 3, and Video 4 show examples of the BC formation observed in vitro in LifeAct-EGFP expressing differentiating hepatoblasts. Video 5 shows a 3D reconstruction of EM serial sections of a bile canaliculus formed in vitro, and Video 6 shows a simplified model based on the 3D reconstruction. Video 7 and Video 8 show the formation of hepatocyte lumina in Rab35 knock-down conditions. Video 9 and Video 10 show 3D reconstructions of the embryonic liver tissue injected with LNP-siLuc or LNP-siRab35. Video 9 shows the 3D reconstructed luminal network, and Video 10 focuses on the organization of the cells forming the lumina. Table S1 provides a list of candidate genes in the focused siRNA screen, and Table S2 provides the sequences of the used siRNAs.

## Supplementary Material

Table S1 shows genes included in the focused siRNA screen.

Table S2 shows siRNA sequences used in the study.

## Data Availability

Bulk RNA-seq data were deposited in GEO under accession no. GSE176069. Codes for the scripts are available upon request. All unique/stable reagents generated in this study are available from the corresponding author with a completed Materials Transfer Agreement.
