# Supplementary material for: Anisotropic expansion of hepatocyte lumina enforced by apical bulkheads
Source: J Cell Biol. 2021 Jul 30;220(10):e202103003. doi: 10.1083/jcb.202103003 (PMC8329733; doi:10.1083/jcb.202103003)
Supplement: Table S1 — shows genes included in the focused siRNA screen. [file JCB_202103003_TableS1.docx]

**Table 1: Genes included in the focused siRNA screen**

| **Gene name** | **Gene ID** | **Lumen phenotype** | **Number of siRNAs with phenotype** | **Category** |
| --- | --- | --- | --- | --- |
| *Tjp1* | 21872 | Loss of polarity | 4/6 | Apical junctions |
| *Ocln* | 18260 | Loss of polarity | 2/6 | Apical junctions |
| *Cldn2* | 12738 | None | 0/6 | Apical junctions |
| *Gjb1* | 14618 | None | 0/6 | Apical junctions |
| *Pard3* | 93742 | None | 0/6 | Apical junctions/ Cytoskeleton |
| *Mark2 (Par1b)* | 13728 | None | 0/6 | Cytoskeleton |
| *Kif13b* | 16554 | None | 0/6 | Cytoskeleton |
| *Stk11 (Lkb1)* | 20869 | None | 0/6 | Cytoskeleton |
| *Cdc42* | 12540 | Spherical lumina | 3/6 | Cytoskeleton/ Polarized trafficking |
| *Arf6* | 11845 | Cyst-like lumina | 1/6 | Cytoskeleton/ Polarized trafficking |
| *Rab35* | 77407 | Cyst-like lumina | 5/6 | Cytoskeleton/ Polarized trafficking |
| *Rab4a* | 19341 | None | 0/6 | Polarized trafficking |
| *Rab4b* | 19342 | None | 0/6 | Polarized trafficking |
| *Rab8a* | 17274 | None | 0/6 | Polarized trafficking |
| *Rab8b* | 235442 | None | 0/6 | Polarized trafficking |
| *Rab10* | 19325 | None | 0/6 | Polarized trafficking |
| *Rab11a* | 53869 | None | 0/6 | Polarized trafficking |
| *Rab11b* | 19326 | None | 0/6 | Polarized trafficking |
| *Rab13* | 68328 | None | 0/6 | Polarized trafficking |
| *Rab14* | 68365 | None | 0/6 | Polarized trafficking |
| *Rab17* | 19329 | None | 0/6 | Polarized trafficking |
| *Rab21* | 216344 | None | 0/6 | Polarized trafficking |
| *Rab27a* | 11891 | None | 0/6 | Polarized trafficking |
| *Rab27b* | 80718 | None | 0/6 | Polarized trafficking |
| *Mal2* | 105853 | None | 0/6 | Polarized trafficking |
